# Supplementary material for: Comparative Analysis of Patient-Matched PDOs Revealed a Reduction in OLFM4-Associated Clusters in Metastatic Lesions in Colorectal Cancer
Source: Stem Cell Reports. 2021 Mar 11;16(4):954–67. doi: 10.1016/j.stemcr.2021.02.012 (PMC8072036; doi:10.1016/j.stemcr.2021.02.012)
Supplement: Document S1. Supplemental Experimental Procedures and Figures S1–S7 [file mmc1.pdf]

Stem Cell Reports, Volume *16*

Supplemental Information

Comparative Analysis of Patient-Matched PDOs Revealed a Reduction in OLFM4-Associated Clusters in Metastatic Lesions in Colorectal Cancer

Takuya Okamoto, David duVerle, Katsuyuki Yaginuma, Yasuko Natsume, Hitomi Yamanaka, Daisuke Kusama, Mayuko Fukuda, Mayuko Yamamoto, Fanny Perraudeau, Upasna Srivastava, Yukie Kashima, Ayako Suzuki, Yuuta Kuze, Yu Takahashi, Masashi Ueno, Yoshiharu Sakai, Tetsuo Noda, Koji Tsuda, Yutaka Suzuki, Satoshi Nagayama, and Ryoji Yao

Supplemental Information

Comparative analysis of patient-matched PDOs revealed the reduction of OLFM4-associated clusters in metastatic lesions in colorectal cancer.

Takuya Okamoto, David duVerle, Katsuyuki Yaginuma, Yasuko Natsume, Hitomi Yamanaka, Daisuke Kusama, Mayuko Fukuda, Mayuko Yamamoto, Fanny Perraudeau, Upasna Srivastava, Yukie Kashima, Ayako Suzuki, Yuuta Kuze, Yu Takahashi, Masashi Ueno, Yoshiharu Sakai, Tetsuo Noda, Koji Tsuda, Yutaka Suzuki, Satoshi, Nagayama, Ryoji Yao

Supplementary Figure 1

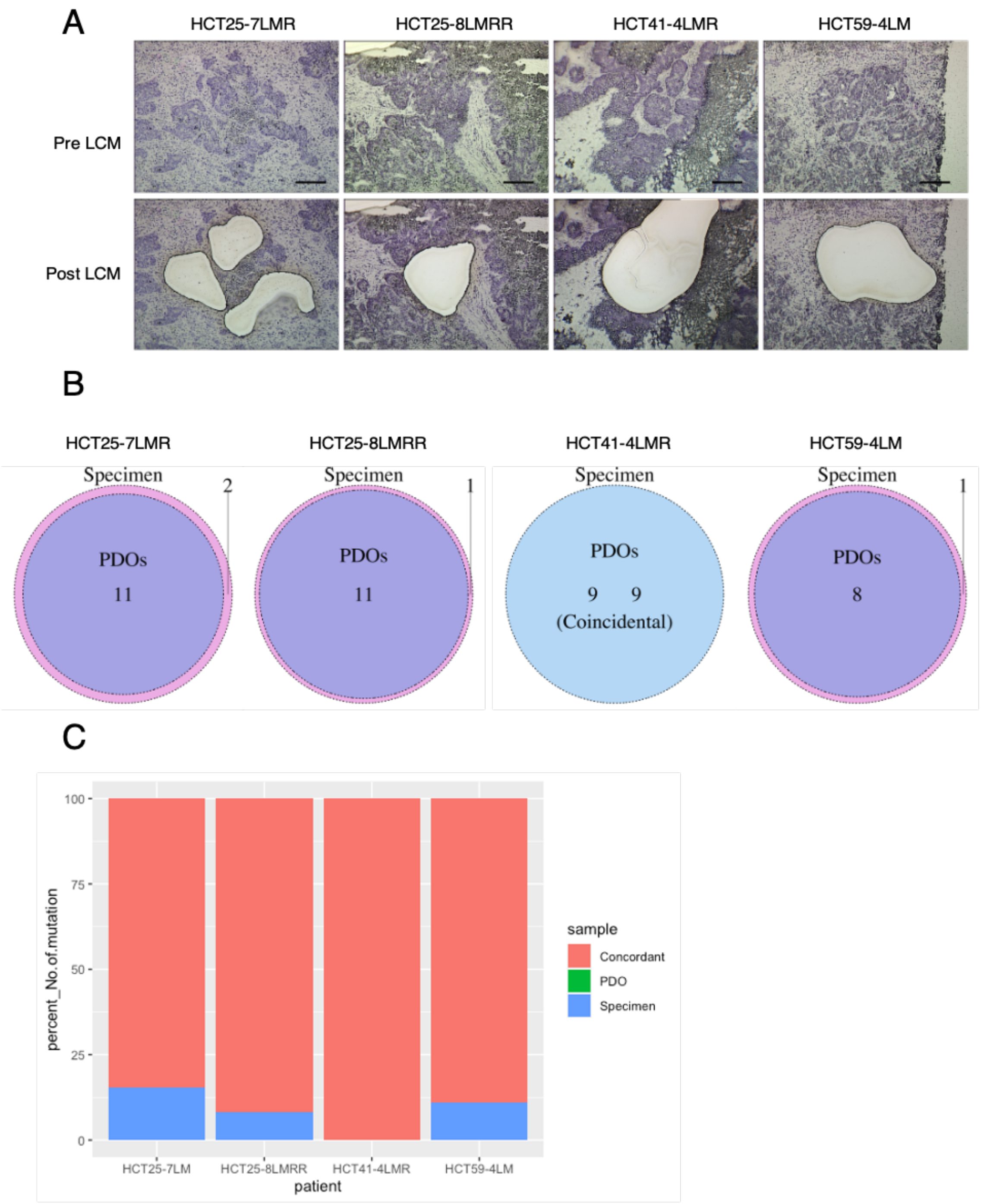

Supplementary Figure 2

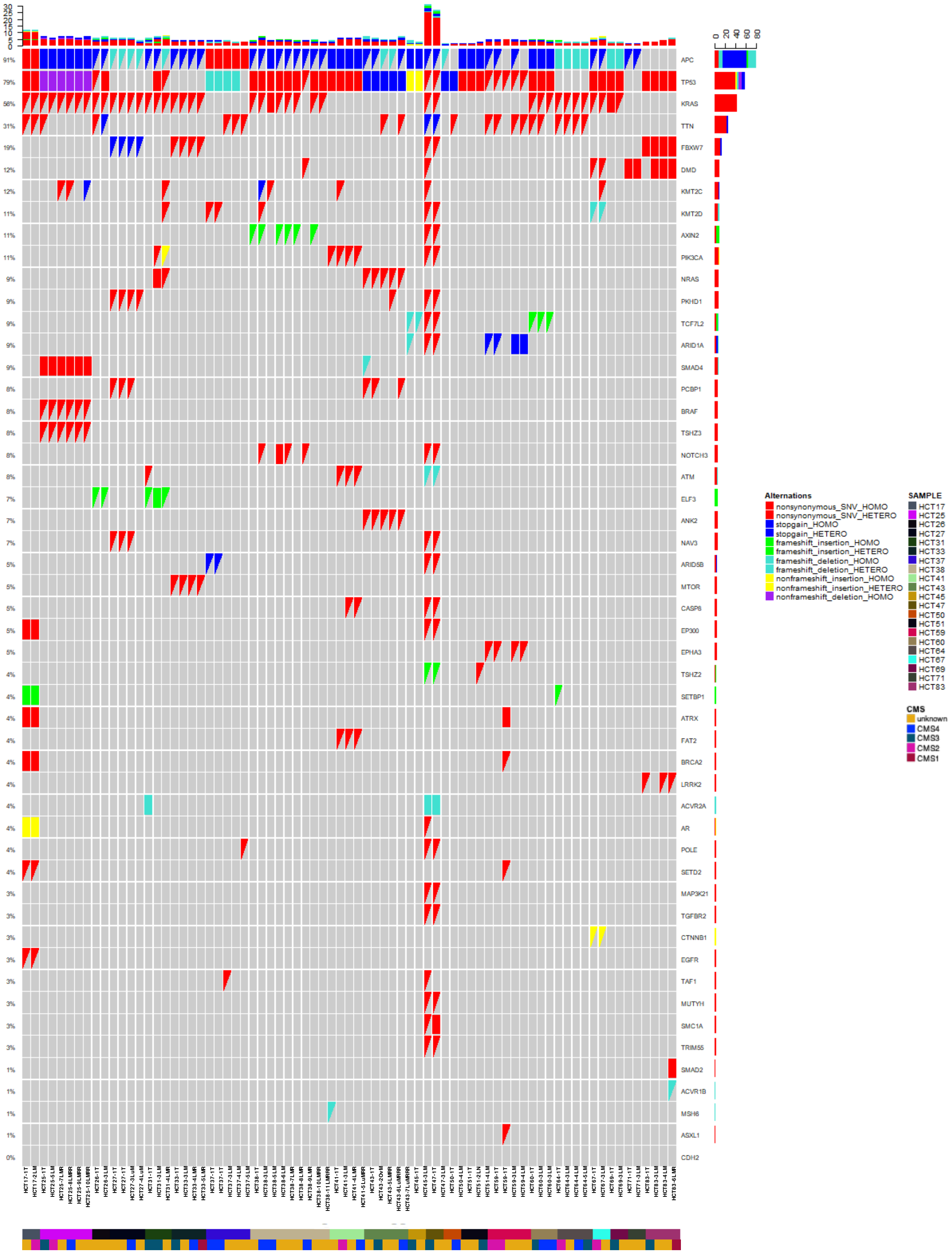

Supplementary Figure 3

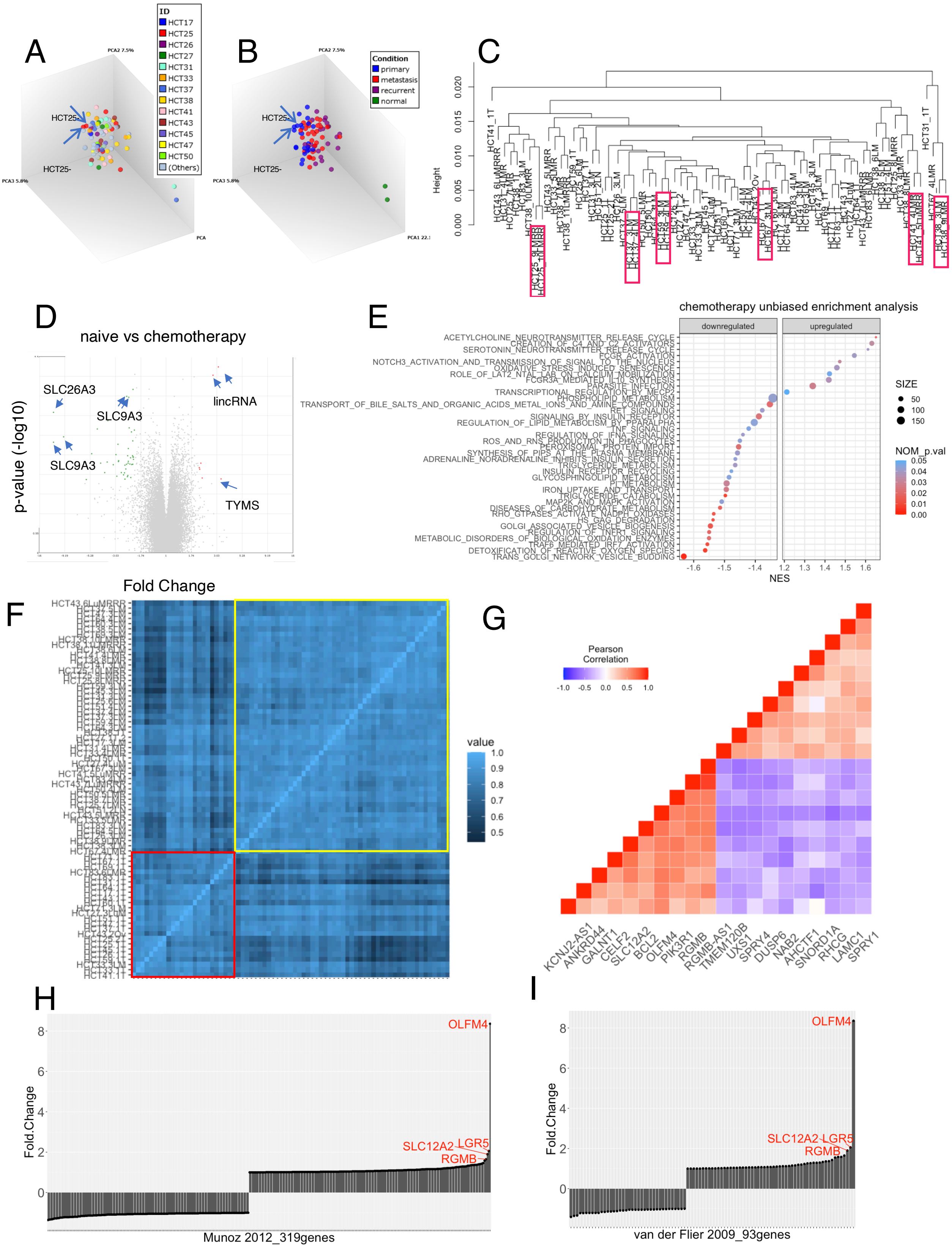

Supplementary Figure 4

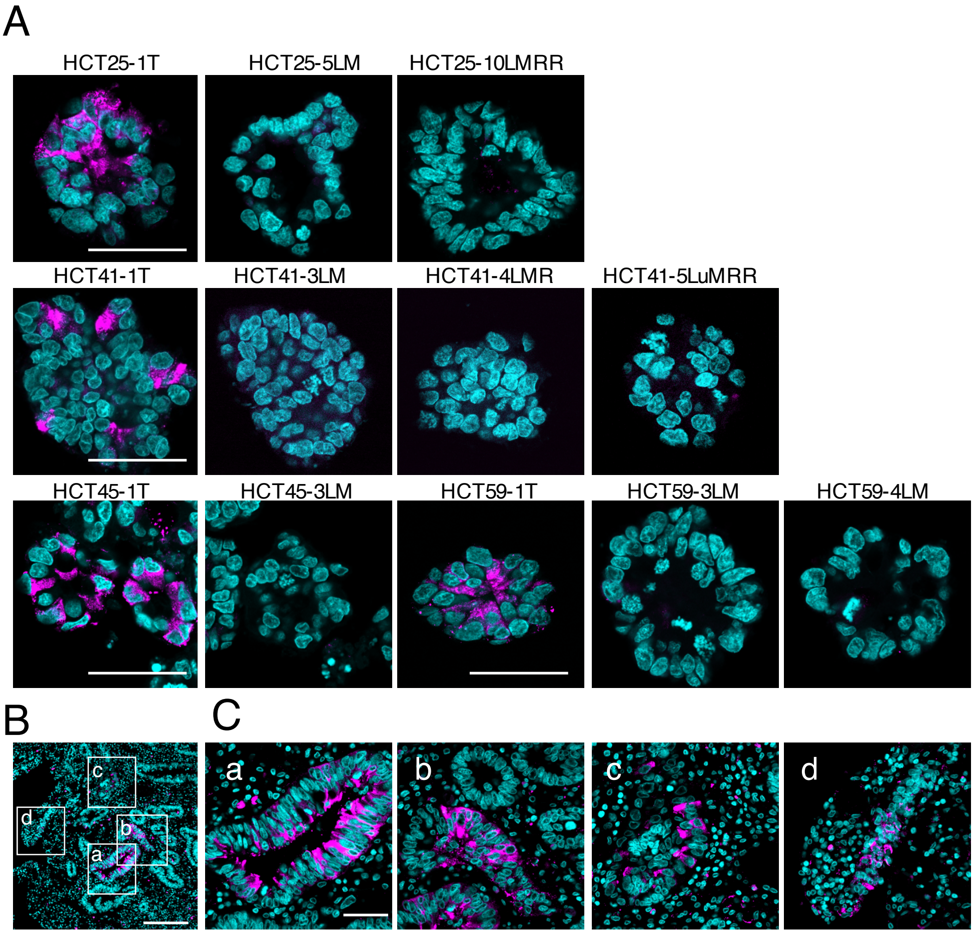

Supplementary Figure 5

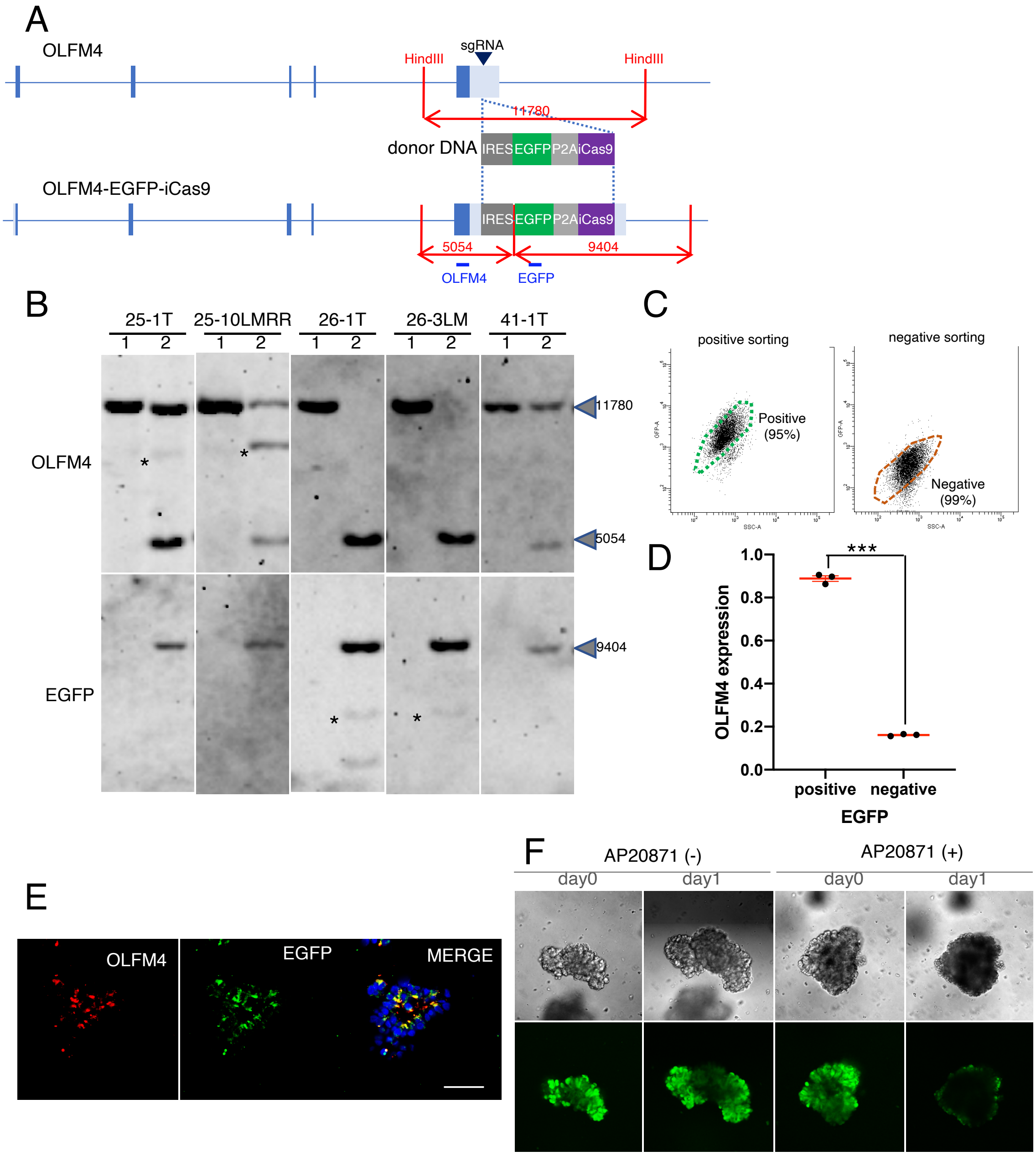

Supplementary Figure 6

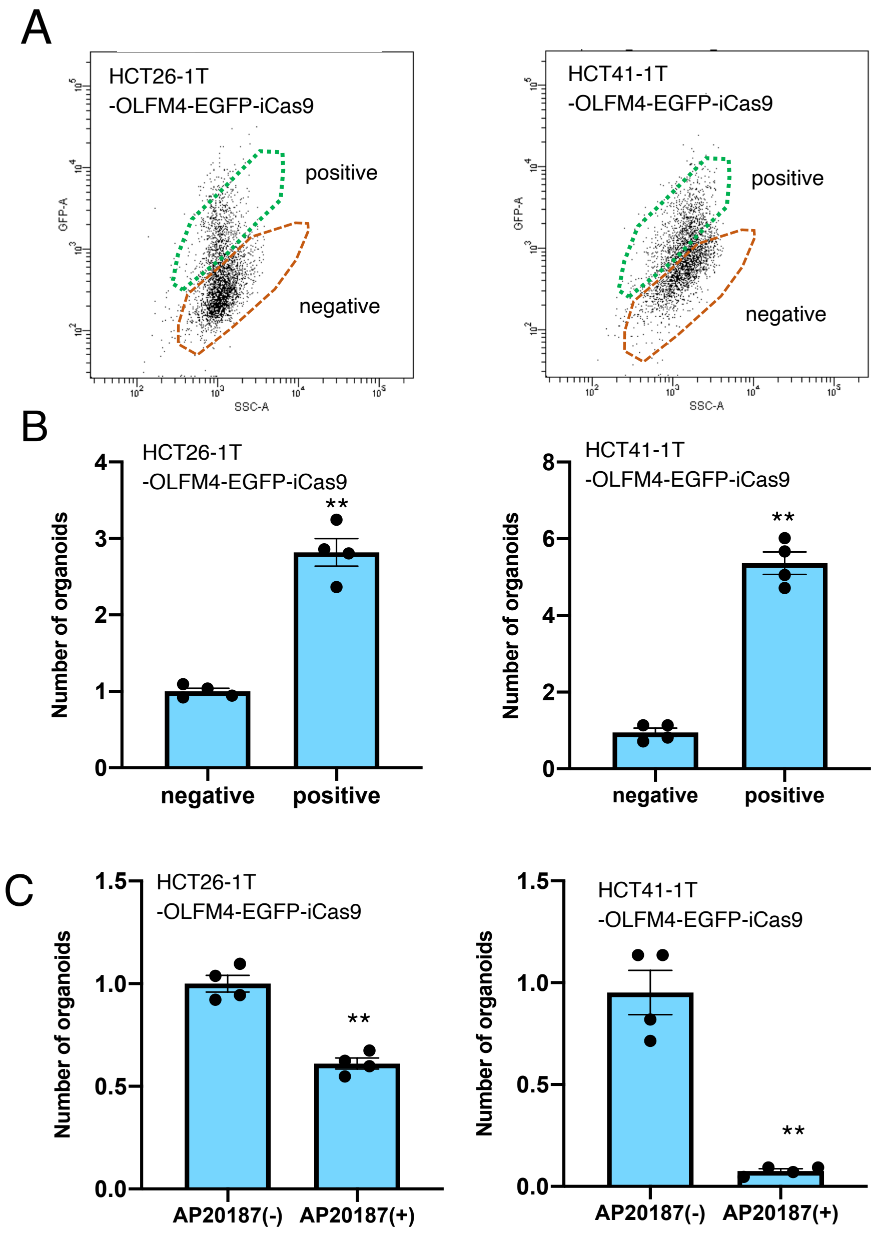

Supplementary Figure 7

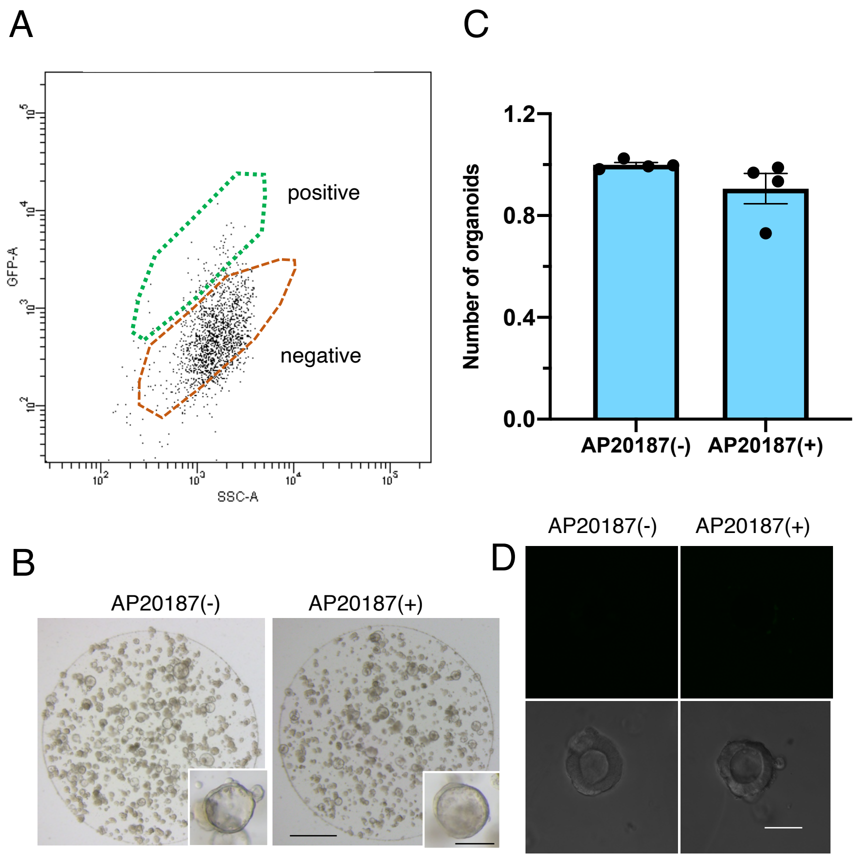

Supplementary Figure Legends

Supplementary Figure 1. Mutations in PDOs and corresponding surgical specimens. Related to Figure 1.

(A) Hematoxylin-stained frozen sections of surgical specimens. Images pre- and post-laser captured microdissection are shown. Bar=200µm

(B) Venn diagram showing the concordance of mutations shared between PDOs and the corresponding surgical specimen.

(C) Concordance of mutations detected in PDOs and corresponding surgical specimens. The bar graph represents the percentage of mutations that are detected only in PDOs (PDOs) and the corresponding surgical specimen (Specimen). Mutations detected in both samples are shown (Concordant).

Supplementary Figure 2. Genomic profiles of CRC PDOs. Related to Figure 1.

Overview of 69 mutations found in 71 PDOs. The nine most frequently mutated genes are listed, and the mutation frequencies are shown in the right row. The patients from which the PDOs were established and their CMS grouping are shown in the bottom column.

Supplementary Figure 3. Transcriptome analysis of PDOs. Related to Figure 1.

(A, B) Principal component analysis of PDOs. Colors represent the patients (A) and lesions (B) from which PDOs were established. Two PDOs established from normal mucosa are shown in green (B). Two primary PDOs derived from identical surgical specimens (HCT25-1T and -2T) are indicated by arrows. (C) Dendrograms (average distance method) based on Pearson correlation. The red boxes show PDO clusters derived from the same patients. (D) Volcano plot of comparative gene expression analysis between naïve and chemotherapy-treated PDOs. PDOs were cultured for five days, and microarray analysis was performed. Red dots represent genes exhibiting a significant difference (fold change > 2.0 and p-val<0.01). (E) Dot plot of GSEA results. The significantly activated and suppressed pathways derived from REACTOME are listed. The color of the dots represents the nominal P value, and the diameter represents the enriched gene count. (F) Correlation matrix of PDOs. The strength of the correlation is represented by color. Pearson's correlation analysis. The clusters mostly composed of

primary PDOs and metastatic/recurrent PDOs are shown by red and yellow frames, respectively. (G) Correlation matrix of gene expression. Ten genes most positively and negatively correlated with OLFM4 are shown in red and blue, respectively. Numbers represent correlation coefficients. (H, I) The expression of ISC signature genes reported by Munoz et al. (H) and van der Filer et al. (I) in PDOs is shown. The differential expression between primary and patient-matched PDOs is indicated by the fold change. The genes included in the metastasis signature are shown in red.

Supplementary Figure 4. Immunohistochemical analysis of OLFM4. Related to Figure 4.

(A) PDOs were fixed, and FFPE sections were stained with an anti-OLFM4 antibody (shown in magenta). Nuclei were visualized using DAPI (shown in cyan). Bar=50 μm. (B, C) Surgical specimens of HCT41-1T were fixed, and FFPE sections were stained with an anti-OLFM4 antibody (shown in magenta). Nuclei were visualized using DAPI (shown in cyan). Bar=200 μm. (C) High magnification of panel B. Bar=50 μm.

Supplementary Figure 5. Genome editing of PDOs. Related to Figures 5 to 7.

(A) An IRES-EGFP-P2A-iCas9 cassette was inserted into the 3' UTR of OLFM4. HindIII sites and the probes used for Southern blot analysis are shown in red and blue, respectively. The expected fragment sizes for each probe are shown in red. (B) Southern blot analysis of genome-edited PDOs. 1. Control PDOs; 2. Genome-edited PDOs. The probes and signal sizes are shown. Single allelic mutant organoids were obtained except in HCT26-1T and 3LM, which harbored biallelic insertions. (C) Fluorescence-activated cell sorting (FACS) chart of HCT25-1T and HCT25-1T cells harboring the IRES-EGFP-P2A-iCaspase9 cassette in the 3' UTR of the OLFM4 locus (HCT25-1T-OLFM4-EGFP-iCas9). The gates for EGFP-positive and EGFP-negative cells are shown in green and red, respectively. (D) RT-PCR analysis of OLFM4. EGFP-positive and EGFP-negative HCT25-1T-OLFM4-EGFP-P2A-iCaspase9 cells were isolated by FACS sorting, as shown in Figure S5C, and OLFM4 expression was analyzed by real-time RT-PCR. The data are shown as the mean and SD (shown in red). *** $P<0.001$ (unpaired t-test, N=3) (E) Immunofluorescence analysis using OLFM4 (shown in red) and EGFP (shown in green). Nuclei were visualized using DAPI. Bar=50 μm. (F) Ablation of $OLFM4^{+}$ cells. HCT25-1T/OLFM4-EGFP-iCas9 organoids were treated

with 2.5 nM AP20871 (AP20871(+)) or left untreated (AP20871(-)) for 1 day. Bar=50 µm.

Supplementary Figure 6. Organoid reconstruction efficiency from single cells. Related to Figure 5-6. (A) Fluorescence-activated cell sorting (FACS) chart of HCT26-1T and HCT41-1T harboring the IRES-EGFP-P2A-iCaspase9 cassette in the 3' UTR of the OLFM4 locus (HCT26-1T-OLFM4-EGFP-iCas9 and HCT41-1T-OLFM4-EGFP-iCas9, respectively). The gates for EGFP-positive and EGFP-negative cells are shown in green and red, respectively. (B) Organoid reconstruction efficiency from single cells. Flow-sorted single cells of HCT26-1T-OLFM4-iCas9 or HCT41-1T-OLFM4-iCas9 were cultured for 9 days, and the number of organoids was counted. The number of organoids relative to that of organoids generated from $OLFM4^-$ cells is shown. Data are shown as the mean and SD. **$p<0.01$(unpaired t-test, n=4 independent experiments). (C) AP20187 suppressed the growth of EGFP-negative cells. Flow-sorted EGFP-negative HCT26-1T-OLFM4_EGFP-iCas9 or HCT41-1T-OLFM4-iCas9 cells were cultured with or without AP20187 for 9 days. The number of organoids was counted and is shown as the relative number of organoids generated without AP20187. Data are shown as the mean and SD. **$p<0.01$(unpaired t-test, n=4 independent experiments).

Supplementary Figure 7. Generation of organoids from $OLFM4^-$ single cells in metastatic PDOs. Related to Figure 7. (A) FACS chart of HCT26-3LM-OLFM4-EGFP-iCas9. The gates for EGFP-positive and EGFP-negative cells are shown in green and red, respectively. (B) Reconstitution of organoids from EGFP-negative cells without (-) or with (+) AP20187. Bar=1000 µm, bar in inset=200 µm (C) The efficiency of organoid reconstitution. The number of organoids was counted using Fiji software, and the number of organoids when cultured with AP20187 relative to the number of organoids generated without AP20187 is shown. Data are shown as the mean and SD. **$p<0.01$(unpaired t-test, n=4 independent experiments). (D) Lack of EGFP expression in metastatic PDOs. Organoids were cultured for seven days and analyzed under a confocal microscope. Bar=50 µm.

Table S1. Clinical information of PDOs derived from stage IV CRC.

Table S2. Genes differentially expressed among distinct lesions.

Table S3. Expression of metastatic signature genes in PDOs derived from primary tumors and the patient matched metastatic lesions.

Table S4. Expression of ISC signatures (319 genes reported by Munoz et al. 2012) in PDOs derived from primary tumors and the patient matched metastatic lesions.

Table S5. Expression of ISC signatures (93 genes reported by van der Flier et al. 2009) in PDOs derived from primary tumors and the patient matched metastatic lesions.

Table S6. Top200 Differentially expressed genes in five clusters identified by scRNA-seq of CRC PDOs.

Table S7. Oligonucleotides used in this study.

Supplemental Experimental Procedures

Establishment of PDOs. Tumor samples were obtained from patients who provided informed consent, and all procedures were approved by the Research Ethics Board at the JFCR Cancer Institute (Tokyo, Japan). PDOs were established as previously described (Sakahara et al., 2019). Briefly, following surgical resection, tumors were enzymatically dissociated in digestion buffer (DMEM (Thermo Fisher Scientific) containing 0.0625% collagenase (Sigma-Aldrich), 0.125% dispase (Thermo Fisher Scientific) and 2.5% FBS and suspended in Matrigel (BD Bioscience), and 25 μl of this mixture was dispensed into each well of a culture plate. The organoids were cultured in ENR medium (basal medium supplemented with ENR (10 ng/ml EGF (Invitrogen), 10% Noggin conditioned medium and 1 μg/ml R-spondin-1 (R&D system)) at 37°C in 5% O_2.

Sequence analysis. Organoids were collected in Cell Recovery Solution (Corning). To analyze the sequence of surgical specimens, frozen tumor tissue sections were stained with hematoxylin, and tumor regions were excised by laser capture microdissection using a Leica LMD6000 microscope. Genomic DNA was extracted with a QIAamp DNA Mini Kit (Qiagen), and the DNA quality was analyzed using the Qubit system (Thermo Fisher Scientific). Two hundred nanograms of genomic DNA was used for library construction with a HaloPlex Kit (Agilent) according to the manufacturer's instructions. Target genes were selected as previously reported (Sakahara et al., 2019). Sequencing was performed using a MiSeq system (Illumina). Single-nucleotide variants (SNVs) and indels were called by the GATK (version 4.0.3) HaplotypeCaller (DePristo et al., 2011). The variants were filtered using GATK VariantFiltration with the following parameters: for SNV: --filter-expression "QD < 2.0 || FS > 60.0 || MQ < 40.0 || HaplotypeScore > 13.0 || MappingQualityRankSum < -12.5 || ReadPosRankSum < -8.0", for indel: --filter-expression "QD < 2.0 || FS > 200.0 || ReadPosRankSum < -20.0". The filtered variants were annotated with ANNOVER (Wang et al., 2010). After annotation, we extracted nonsynonymous SNVs and indels using our Perl script.
To extract somatic mutations, we excluded germline variants of NCBI dbSNP build 147 (Sherry et al., 2001) from the detected variants of tumor tissues and rescued known somatic mutations registered in the COSMIC database (version 70) (Forbes et al., 2017)

using our in-house Perl script. We also removed germline variants that were detected in normal tissue samples.

Microarray analysis. Organoids were collected in Matrigel Recovery Solution (Corning), and RNA was extracted using an RNeasy Micro Kit (Qiagen). RNA quality was validated using an Agilent Bioanalyzer. One hundred nanograms of RNA was used to prepare cRNA with a 3' IVT PLUS Reagent Kit (Affymetrix), and the cRNA was hybridized on the Human Transcriptome Array 2 (HTA2) (Affymetrix). Data were analyzed using Transcriptome Analysis Console (TAC) (Affymetrix). Analysis and visualization of the transcriptome data were performed in R (version 4.0) and RStudio (version 1.2). Clustering was carried out using the average method in the hclust function, and correlation analysis was performed with the Pearson method. The results were visualized using gglot2 (version 3.0.01).
Molecular subtyping was performed on the microarray transcriptome data in R (version 3.5.1) using the oligo package (version 1.46.0). After normalization using the RMA function, classification was performed using the CMScaller method with the default parameters to classify the CMS function (Eide et al., 2017).

Histochemical analysis. PDOs cultured in Matrigel for four to 6 days were extracted with Matrigel Recovery Solution, washed with PBS, fixed in 4% paraformaldehyde (PFA), held in 70% ethanol, and embedded in HitoGel (Thermo Fisher Scientific) before paraffin embedding. After dewaxing and rehydration via a standard procedure, antigen retrieval was performed by autoclaving in citrate buffer (pH 6.0) or by microwave treatment in TE buffer (pH 8.0). Surgical specimens were fixed in formalin and embedded in paraffin; the slides were incubated overnight at 4°C with primary anti-OLFM4 antibodies (D1E4M, Cell Signaling #14369) followed by Cy3-conjugated secondary antibodies (Merck Millipore, APC132).

scRNA-seq. PDOs were dissociated with Triple LE Express (Life Technology); undigested cell clusters were removed with a 40-μm cell strainer (Falcon). Single cells were washed twice with PBS. ScRNA-seq libraries were prepared using Chromium Single Cell 3' Solution (Reagent Kits version 1, 10x Genomics) according to the manufacturer's instructions. Briefly, using a Chromium Single Cell 3' Library, Gel

Bead & Multiplex Kit and Chip Kit (10x Genomics), Gel Bead-In-EMulsions (GEMs) constructed from isolated cells, Master Mix, Gel Beads and Partitioning Oil were created using the Chromium system (10x Genomics). After GEM generation, GEM-RT was performed, and the barcoded cDNA was cleaned and amplified. The amplified cDNAs were quantified using an Agilent 2100 Bioanalyzer High Sensitivity DNA Kit (Agilent). The cDNAs were sheared and size-selected for library construction; end repair, A-tailing, adaptor ligation and sample index PCR were also conducted. The constructed library was quantified using a Bioanalyzer.

The libraries were sequenced using a HiSeq2500 system (Illumina) according to the manufacturer's instructions. After sequencing analysis, fastq files were created using Cell Ranger (10x Genomics).

We filtered out cells that were 3x the mean absolute deviation (MAD) from the mean of the log coverage or 2x the MAD of the percentage of zeros. Genes without 1 count among at least 10 cells were filtered out, resulting in the removal of 4763 genes. Sample bias was corrected using the zero-inflated negative binomial model (ZINB-WaVE) (Risso et al., 2018).

Genome editing and in vitro analysis of PDOs. We used CRISPR/Cas9-mediated homology-independent targeted integration (HITI) to achieve efficient genome editing (Suzuki et al., 2016). sgRNA-expressing constructs were prepared by cloning a pair of annealed oligonucleotides into the BbsI site of pX330 (Addgene #422320). To construct a donor vector for OLFM4-EGFP-P2A-iCaspase9, the P2A-iCaspase9 fragment was amplified by PCR using pMSCV-F-del Casp9.IRES. GFP (Addgene #15567) was used as a template, and PvuI and PacI sites were created. The fragment was then cloned into the PacI site of pCMMP-MCS-IRES-eGFP (Addgene #36953), which was modified to disrupt the stop codon of the eGFP gene. The 20-bp sgRNA recognition sequence oligonucleotide was cloned into AgeI/NotI and PacI/NheI sites. The oligonucleotide sequences are listed in Table S7. The donor construct and sgRNA and Cas9 expression vector were electrophoresed using the piggyBac system as previously described (Fujii et al., 2015). The transfected organoids were selected with 2 μg/ml puromycin from 5 days after electroporation. Genomic DNA was prepared using lysis buffer (10 mM Tris-HCl, pH 8.0, 25 mM EDTA, 1% SDS, 150 mM NaCl, 100 μg/ml Proteinase K), followed by phenol-chloroform extraction and ethanol precipitation. Southern blot analysis was

performed using standard procedures and the DIG system (Roche). The primers used to prepare DIG probes are listed in Table S7.

For FACS analysis, PDOs were dissociated using Triple LE Express (Life Technology), and undigested cell clusters were removed with a 20-µm cell strainer (Falcon). The cells were washed twice with PBS supplemented with 0.2% BSA and 2 mM EDTA and stained with 7-aminoactinomycin D (BD Bioscience). Single cells were gated based on the SSC-H vs SSC-W profile. The cells were sorted using a 100-µm nozzle (Aria III, BD Bioscience), and $1x10^4$ cells were embedded in 25 µl of Matrigel and cultured in a 48-well plate. Images were analyzed using the cell counter plugin installed in Image J (version 2.0.0). Data are presented as the mean and SD (error bars) of four independent experiments. Expression of EGFP was analyzed via confocal scanning microscopy (Zeiss LSM880). To evaluate the reconstruction efficiency, pictures were taken using a stereomicroscope (Leica M165C), and the images were analyzed using the cell counter plugin installed in ImageJ (version 2.0.0). All data were derived from four independent experiments.

Supplemental References

DePristo, M.A., Banks, E., Poplin, R., Garimella, K.V., Maguire, J.R., Hartl, C., Philippakis, A.A., del Angel, G., Rivas, M.A., Hanna, M*., et al.* (2011). A framework for variation discovery and genotyping using next-generation DNA sequencing data. Nat Genet *43*, 491-498.

Eide, P.W., Bruun, J., Lothe, R.A., and Sveen, A. (2017). CMScaller: an R package for consensus molecular subtyping of colorectal cancer pre-clinical models. Scientific reports *7*, 16618.

Forbes, S.A., Beare, D., Boutselakis, H., Bamford, S., Bindal, N., Tate, J., Cole, C.G., Ward, S., Dawson, E., Ponting, L*., et al.* (2017). COSMIC: somatic cancer genetics at high-resolution. Nucleic Acids Res *45*, D777-D783.

Fujii, M., Matano, M., Nanki, K., and Sato, T. (2015). Efficient genetic engineering of human intestinal organoids using electroporation. Nat Protoc *10*, 1474-1485.

Risso, D., Perraudeau, F., Gribkova, S., Dudoit, S., and Vert, J.P. (2018). A general and flexible method for signal extraction from single-cell RNA-seq data. Nature communications *9*, 284.

Sakahara, M., Okamoto, T., Oyanagi, J., Takano, H., Natsume, Y., Yamanaka, H., Kusama, D., Fusejima, M., Tanaka, N., Mori, S.*, et al.* (2019). IFN/STAT signaling controls tumorigenesis and the drug response in colorectal cancer. Cancer Sci.

Sherry, S.T., Ward, M.H., Kholodov, M., Baker, J., Phan, L., Smigielski, E.M., and Sirotkin, K. (2001). dbSNP: the NCBI database of genetic variation. Nucleic Acids Res *29*, 308-311.

Suzuki, K., Tsunekawa, Y., Hernandez-Benitez, R., Wu, J., Zhu, J., Kim, E.J., Hatanaka, F., Yamamoto, M., Araoka, T., Li, Z.*, et al.* (2016). In vivo genome editing via CRISPR/Cas9 mediated homology-independent targeted integration. Nature *540*, 144-149.

Wang, K., Li, M., and Hakonarson, H. (2010). ANNOVAR: functional annotation of genetic variants from high-throughput sequencing data. Nucleic Acids Res *38*, e164.
