# Supplementary material for: Comparative Analysis of Patient-Matched PDOs Revealed a Reduction in OLFM4-Associated Clusters in Metastatic Lesions in Colorectal Cancer
Source: Stem Cell Reports. 2021 Mar 11;16(4):954–67. doi: 10.1016/j.stemcr.2021.02.012 (PMC8072036; doi:10.1016/j.stemcr.2021.02.012)
Supplement: Document S2. Article plus Supplemental information [file mmc9.pdf]

Stem Cell Reports
Resource
OPEN ACCESS

Comparative Analysis of Patient-Matched PDOs Revealed a Reduction in OLFM4-Associated Clusters in Metastatic Lesions in Colorectal Cancer

Takuya Okamoto,[1,2] David duVerle,[3] Katsuyuki Yaginuma,[1] Yasuko Natsume,[1] Hitomi Yamanaka,[1] Daisuke Kusama,[1] Mayuko Fukuda,[1] Mayuko Yamamoto,[1] Fanny Perraudeau,[4] Upasna Srivastava,[3] Yukie Kashima,[3] Ayako Suzuki,[3] Yuuta Kuze,[3] Yu Takahashi,[5] Masashi Ueno,[5] Yoshiharu Sakai,[2] Tetsuo Noda,[6] Koji Tsuda,[3] Yutaka Suzuki,[3] Satoshi Nagayama,[5] and Ryoji Yao[1,*]
[1]Department of Cell Biology, Cancer Institute, Japanese Foundation for Cancer Research, Tokyo, Japan
[2]Department of Surgery, Graduate School of Medicine, Kyoto University, Kyoto, Japan
[3]Department of Computational Biology and Medical Sciences, Graduate School of Frontier Sciences, The University of Tokyo, Kashiwa, Chiba, Japan
[4]Graduate Group in Biostatistics, University of California, Berkeley, Berkeley, CA, USA
[5]Department of Gastroenterological Surgery, Cancer Institute Hospital, Japanese Foundation for Cancer Research, Tokyo, Japan
[6]Director's Room, Cancer Institute, Japanese Foundation for Cancer Research, Tokyo, Japan
*Correspondence: ryao@jfcr.or.jp
https://doi.org/10.1016/j.stemcr.2021.02.012

SUMMARY

Metastasis is the major cause of cancer-related death, but whether metastatic lesions exhibit the same cellular composition as primary tumors has yet to be elucidated. To investigate the cellular heterogeneity of metastatic colorectal cancer (CRC), we established 72 patient-derived organoids (PDOs) from 21 patients. Combined bulk transcriptomic and single-cell RNA-sequencing analysis revealed decreased gene expression of markers for differentiated cells in PDOs derived from metastatic lesions. Paradoxically, expression of potential intestinal stem cell markers was also decreased. We identified OLFM4 as the gene most strongly correlating with a stem-like cell cluster, and found OLFM4$^+$ cells to be capable of initiating organoid culture growth and differentiation capacity in primary PDOs. These cells were required for the efficient growth of primary PDOs but dispensable for metastatic PDOs. These observations demonstrate that metastatic lesions have a cellular composition distinct from that of primary tumors; patient-matched PDOs are a useful resource for analyzing metastatic CRC.

INTRODUCTION

Tumor tissues consist of functionally heterogeneous cells (Engel et al., 2020; Magee et al., 2012; Meacham and Morrison, 2013). These cells are organized into subpopulations of stem-like cells and their differentiated progeny, which often correspond to the composition, hierarchy, and cell fate behavior of the corresponding normal tissues. In mouse, Lgr5 expression marks stem cells in normal small intestine and tumor tissues, demonstrating that tumor tissues consist of heterogeneous cells mirroring the cellular hierarchy of the normal intestine (Barker, 2014; Barker et al., 2007; Clevers, 2013; Schepers et al., 2012). Recent comparative analysis of human normal colon and tumor tissue demonstrated similar but less variable cellular heterogeneity (Li et al., 2017).

Metastases account for the majority of cancer-associated deaths, and cellular heterogeneity is considered to be critical for the development of metastasis (Bedard et al., 2013; Birkbak and McGranahan, 2020; Meacham and Morrison, 2013; Tieng et al., 2020). Because metastatic tissues arise from disseminated tumor cells from primary sites, their potential to colonize distant organs and to generate metastatic tumors may be attributed to the competence of stem cells in primary tumors. However, the process of metastasis consists of multiple steps, and distinct cellular functions are required for each step (Lambert et al., 2017; Massague and Obenauf, 2016). Comprehensive analyses of genetic alterations that differentiate metastatic from primary lesions have been carried out, leading to the notion that most somatic mutations are present in both the primary tumor and paired metastasis (Ishaque et al., 2018; Schweiger et al., 2018; Xie et al., 2014). Transcriptome analyses have revealed highly similar gene expression patterns between primary tumors and metastatic lesions (Lee et al., 2016; Vignot et al., 2015). These analyses have deepened our understanding of metastasis, but whether metastatic lesions recapitulate the cellular composition of primary tumors remains elusive.

Patient-derived organoids (PDOs) recapitulate many aspects of the clinical features of original tumors, including genetic alterations and the gene expression profile (Boj et al., 2015; Engel et al., 2020; Fujii et al., 2016; Huang et al., 2015; Karthaus et al., 2014; Nanki et al., 2018; Ooft et al., 2019; Sachs et al., 2018; van de Wetering et al., 2015; Vlachogiannis et al., 2018; Weeber et al., 2015). PDOs also retain the cellular composition of the corresponding tissue (Cortina et al., 2017; Sato et al., 2009; Shimokawa et al., 2017). Lineage tracing of PDOs has recently been reported, demonstrating that LGR5$^+$ cells possess self-renewal and differentiation capacity. Notably, LGR5$^-$ cells can produce LGR5$^+$ cells and contribute to tumor regrowth after LGR5$^+$ cell ablation (Shimokawa et al., 2017), indicating cellular plasticity. These technological advances provide an opportunity to analyze

This is an open access article under the CC BY license (http://creativecommons.org/licenses/by/4.0/).

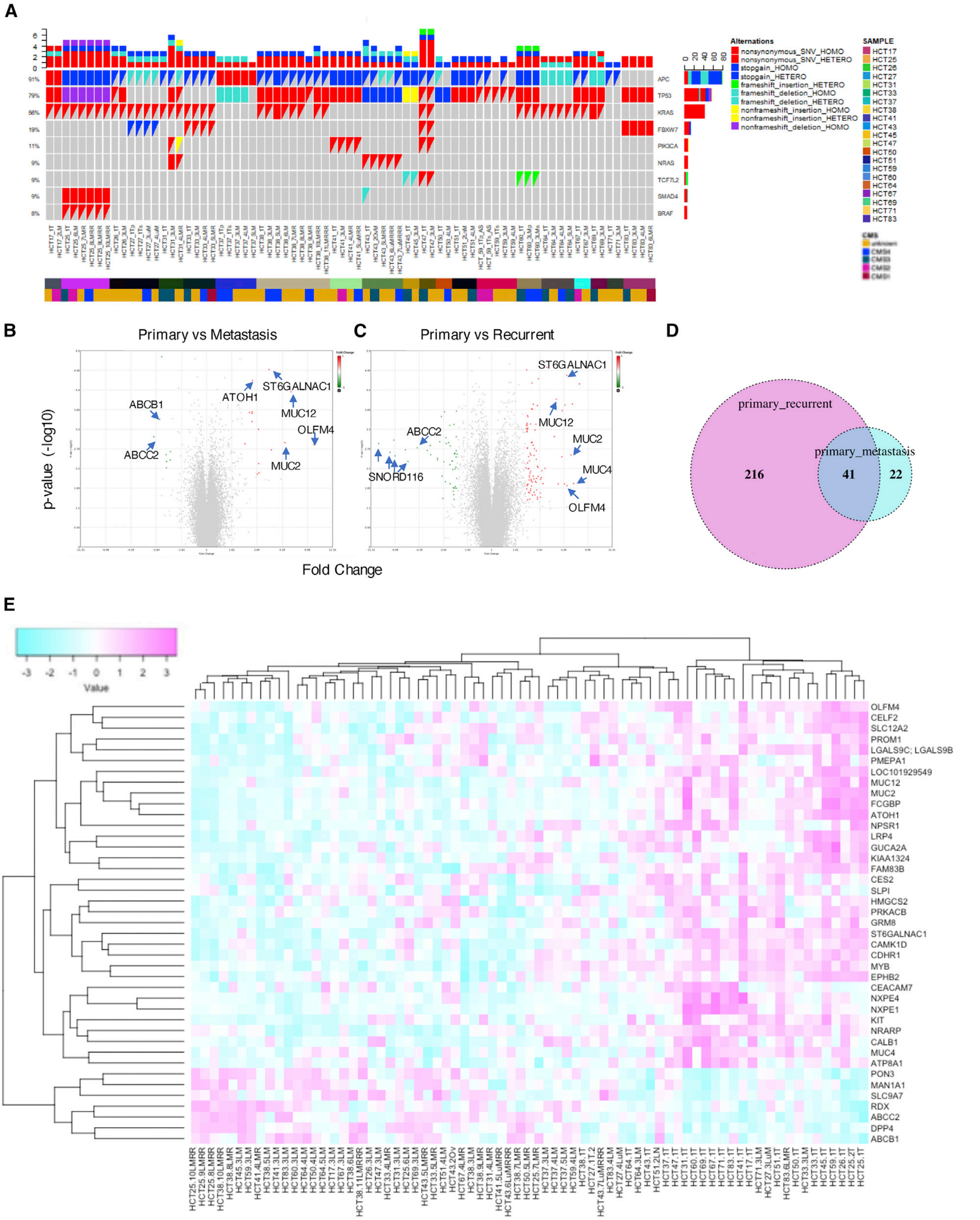

(legend on next page)

cellular heterogeneity in identical culture conditions and to validate its functional significance.

RESULTS

Establishment of Patient-Matched CRC PDOs

We established PDOs from primary tumors and patient-matched metastatic lesions from 21 stage IV CRC patients. The clinical characteristics of the patients, including sex, age, and tumor location, are presented in Table S1. Among them, single metastatic organoids were obtained for 14 cases, and multiple metastatic organoids derived from independent lesions were obtained for 7 cases. The metastasis sites included the liver (27), lung (2), ovary (1), and lymph node (1). All patients were followed-up after the initial surgery, and additional PDOs were established from recurrent tumors. As a result, 19 PDOs were established from recurrent tumors, including 16 liver and 3 lung lesions. Seven patients received preoperative chemotherapy, and 17 PDOs were established. In total, 72 PDOs were used in this study.

We performed targeted sequencing of 69 recurrently mutated genes in CRC (Sakahara et al., 2019). To examine whether the PDOs recapitulated the mutation profile of the original tumors, four laser capture microdissection samples from frozen specimens were analyzed (Figure S1). The mutations were highly concordant (85%–100%), with four mutations found specifically in the tumor samples. All mutations found in the PDOs were identified in the tumor samples. These highly concordant mutation profiles suggest that the PDOs recapitulated the characteristics of the original tumor tissues. These observations were consistent with those of previous studies of PDOs derived from CRC (Fujii et al., 2016; van de Wetering et al., 2015; Weeber et al., 2015).

Frequently mutated genes in CRC were detected in both primary PDOs and metastatic PDOs (Figures 1 and S2), including APC (91%), TP53 (79%), KRAS (56%), FBXW7 (19%), PIK3CA (11%), NRAS (9%), TCF7L2 (9%), SMAD4 (9%), and BRAF (8%). The mutation frequencies of each gene were consistent with previously reported values (Ishaque et al., 2018). Overall, of 148 mutations, 108 mutations (73%) were detected in the primary tumor and paired metastases, suggesting common progenitor clones in primary sites. In addition, 14 mutations (9.5%) were detected only in primary tumors, whereas 25 mutations (17.6%) were identified as metastasis-specific mutations, confirming the results of previous analysis (Ishaque et al., 2018). These findings suggest that the mutation profiles of PDOs represent those of the corresponding tumors. No recurrent mutations specific to metastasis or chemotherapy-treated PDOs were detected in our cohort, although further comprehensive analysis is needed to evaluate the contribution of gene alterations to metastasis or chemosensitivity.

Transcriptome Analysis of PDOs

Bulk gene expression profiles of PDOs were obtained using microarray analysis. To analyze the expression profile of each PDO, consensus molecular subtypes (CMS) classification was applied to all PDOs (Figure 1A) (Eide et al., 2017). Overall, 2.7%, 9.3%, 17.3%, and 18.7% of the PDOs were classified as CMS1, -2, -3, and -4, respectively. These percentages were comparable with those of clinical samples (Schlicker et al., 2020). However, it is important to note that CMS4 in the CMS classifier used to analyze clinical samples represents a gene profile compatible with stromal infiltration, which reflects higher admixture with mesenchymal cells (Guinney et al., 2015). CMScaller, used to analyze PDOs, is designed to identify intrinsic features of cancer cells (Eide et al., 2017) because their culture system is devoid of mesenchymal cells. Modified culture systems that allow the growth of mesenchymal cells in tumor tissue may provide insight into the correlation between tumor tissues and PDOs at the transcriptional level.

A set of PDOs, namely, HCT25-1T and -2T, which were independently established from the same surgical specimens, exhibited a strong association in principal-component analysis, suggesting that the global expression profile of PDOs was maintained (Figures S3A and S3B). Two organoids derived from the normal mucosa of patients

Figure 1. Genomic and Transcriptome Analyses of PDOs

(A) Genomic profiles of CRC PDOs. Overview of mutations found in 72 PDOs. The nine most frequently mutated genes are listed, and the mutation frequencies are shown in the right row. Patients from whom PDOs were established and their CMS grouping are shown in the bottom column.

(B and C) Volcano plot of comparative gene expression analysis between primary and metastatic (B) and between primary and recurrent (C) PDOs. The PDOs were cultured for 5 to 7 days, and microarray analysis was performed. Red dots represent genes exhibiting a significant expression difference (fold change > 2.0 and $p < 0.01$).

(D) A Venn diagram showing the number of genes common to comparison between primary and metastatic PDOs (shown in blue) and that between metastatic and recurrent PDOs (shown in red).

(E) Heatmap of mRNA expression of metastasis signature genes. Each row was transformed using the *Z* score. The color represents mRNA expression levels scaled across PDOs. Genes and samples were hierarchically clustered using Pearson correlation.

See also Figures S1–S3.

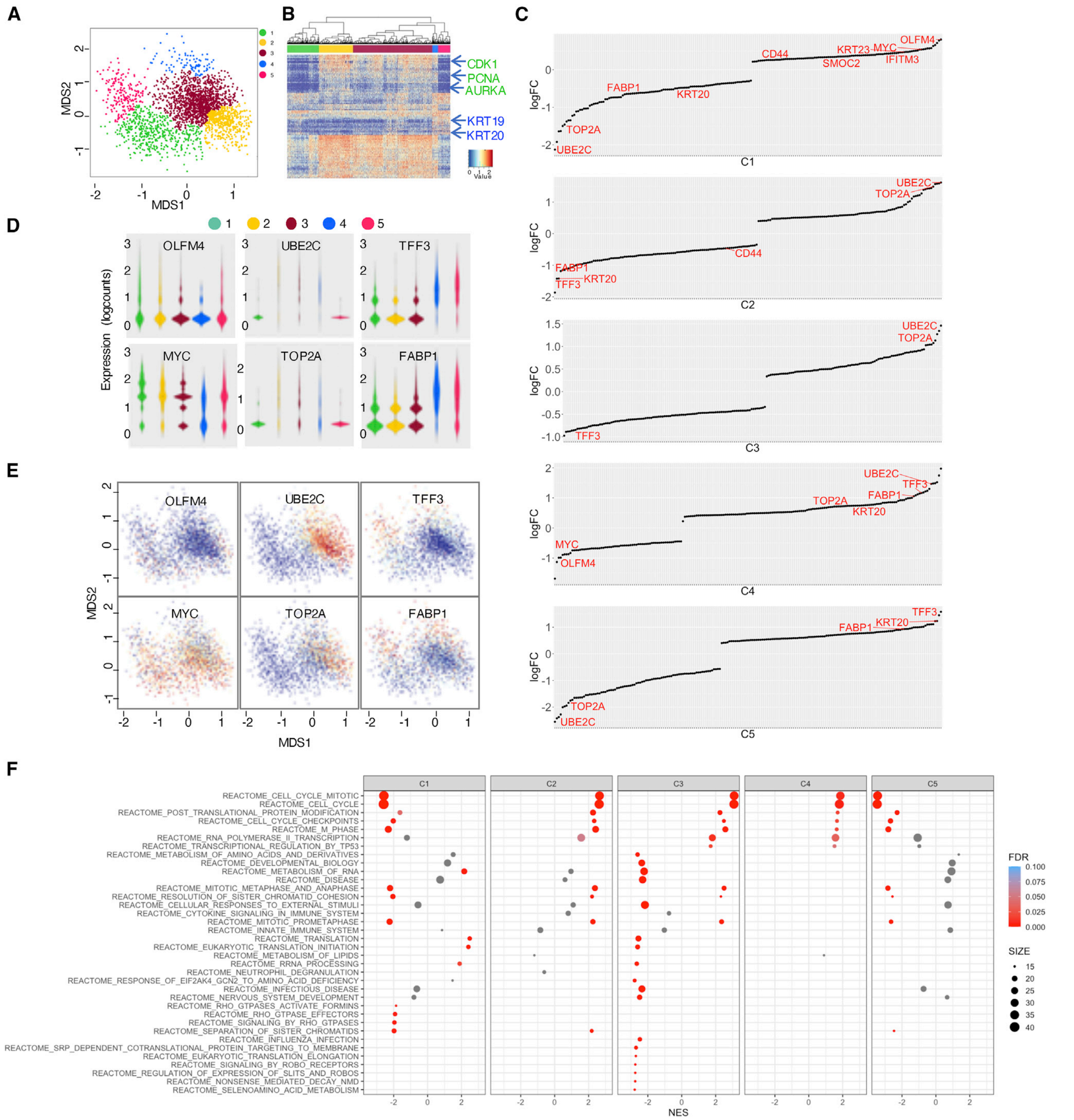

Figure 2. scRNA-Seq Analysis of PDOs

(A) Cell-type clusters. Multiple-dimensional scaling (MDS) was used to visualize the clustering based on the unbiased gene expression analysis. Each cluster is colored.

(B) Cell-type expression signature. A heatmap of the top 100 differentially expressed genes in clusters is shown. Each cluster was tested against all other clusters. Selected gene names are indicated.

(C) Differentially expressed genes in clusters. The top 200 genes are shown by log of fold change (logFC). Selected marker genes for stem cells, proliferating cells, and differentiated cells are shown in red. Genes, logFC, and p values are listed in Table S6.

(D and E) Expression of marker genes in clusters. The distribution of gene expression in each cluster is shown (D). The expression level of the indicated gene is colored tangerine (E).

(legend continued on next page)

HCT31 and -37 included for comparison formed a distinct cluster from the tumor PDOs. Unsupervised clustering of the transcriptome profiles revealed correlations between limited sets of PDOs derived from the same patients (Figure S3C, shown by the red box). Nevertheless, we observed neither a clear separation of primary PDOs from metastatic PDOs nor a homologous clustering of patient-matched PDOs. These exploratory analyses suggest that, despite similar genetic alterations in major driver genes (Figures 1A and S2), the primary PDOs differ from their matched metastatic PDOs at the transcriptome-wide level.

Identification of Genes that Are Differentially Expressed between Primary and Metastatic PDOs

We next searched for genes that were differentially expressed among tumor sites. Patient-matched paired analysis identified 63 genes differentially expressed between primary PDOs and metastatic PDOs (fold change > 1.6, p < 0.05) (Figure 1B). In total, 43 genes were more highly expressed in primary PDOs than in corresponding metastatic PDOs; 20 genes were more highly expressed in metastatic PDOs (Table S2). Among them, OLFM4, which has been reported as a stem cell marker of the human colon (Barker, 2014; van der Flier et al., 2009a), exhibited the most robust difference (fold change = 8.36, p = 0.0017). Paradoxically, higher expression levels of differentiation markers were also noted in primary PDOs, including MUC2 (fold change = 3.48, p = 0.0005) and MUC12 (fold change = 2.41, p = 0.0002). ST6GALNAC1, which catalyzes sialylation of the GalNAC residue on mucins (Ikehara et al., 1999), was also highly expressed in primary PDOs (fold change = 3.33, p = 4.78E−06). The expression level of atonal homolog 1 (Yang et al., 2001), a master transcription factor for secretory lineage differentiation, was significantly higher in primary PDOs than in metastatic PDOs (fold change = 2.42, p = 3.59E−05). These observations suggest that primary PDOs contain a large number of cells of a secretory lineage.

Two ABC transporters, ABCC2 and ABCB1, exhibited the most significant differences (fold change = 2.73, p = 0.0006 and fold change = 2.58, p = 0.0003, respectively). These proteins are involved in the transport of tamoxifen (Iusuf et al., 2011), and polymorphisms in their genes are associated with the risk of metastasis and recurrence in hormone receptor-positive breast cancer (Kiyotani et al., 2010; Sensorn et al., 2013, 2016), suggesting their roles in the metastasis process. Furthermore, overexpression of these genes confers cancer cell resistance to various chemotherapeutic agents (Taniguchi et al., 1996; Xie et al., 2010). Hence, we performed a comparative analysis between naive and chemotherapy-treated PDOs, which identified 248 differentially expressed genes (fold change >1.6, p < 0.05). However, ABCC2 and ABCB1 did not meet the criteria (Figure S3D; Table S2). Gene set enrichment analysis (GSEA) identified 10 and 27 pathways that were overexpressed and underexpressed, respectively, in chemotherapy-treated PDOs (Figure S3E). A larger number of PDOs are needed to elucidate the biological significance of genes highly expressed in metastatic PDOs.

Comparative transcriptome analysis between primary PDOs and patient-matched recurrent PDOs identified 257 differentially expressed genes (fold change > 1.6, p < 0.05) (Figures 1C; Table S2). In an effort to identify genes relevant to both metastatic and recurrent processes, we focused on sets of genes that were differentially expressed in both comparative analyses, and 41 genes met this criterion (Figure 1D; Table S3). Pearson correlation analysis of PDOs using this gene set revealed two major clusters: one mostly composed of primary PDOs (20/25, 80%) (Figure S3F, shown in the red frame) and one composed of metastatic and recurrent PDOs (44/47, 94%) (Figure S3F shows in the yellow frame). Hierarchical clustering demonstrated 34 genes to be highly expressed in primary tumors; seven genes were highly expressed in metastatic lesions (Figure 2E).

Since OLFM4 was included in the set of 41 differentially expressed genes, we next analyzed the expression profiles of two intestinal stem cell (ISC) signatures (Figures S3H and S3I; Tables S4 and S5) (Munoz et al., 2012; van der Flier et al., 2009b). Four genes were significantly downregulated in metastatic PDOs compared with corresponding primary PDOs (fold change > 1.6, p < 0.05) (Figures S3H and S3I). These genes included LGR5 (fold change = 2.06, p = 0.0194), SLC12A2 (fold change = 1.9, p = 0.0003), and RGMB (fold change = 1.65, p = 0.0064). None of the ISC signature genes were significantly upregulated in metastatic PDOs. Interestingly, unbiased Pearson correlation analysis identified three ISC signature genes in the top 10 genes with expression that most correlated with that of OLFM4 (Figure S3G). These included SLC12A2 (coefficient = 0.67), RGMB (coefficient = 0.64), and BCL2 (coefficient = 0.63). LGR5 also correlated positively, although the correlation was less pronounced (coefficient = 0.44). These results suggest that distinct expression of OLFM4 between primary and metastatic PDOs represents altered cell composition rather than a specific effect on OLFM4 expression.

Cellular Composition of CRC PDOs

Bulk gene expression analysis identified potential markers between primary and metastatic/recurrent PDOs. However,

(F) Dot plot of GSEA results. Significantly activated and suppressed pathways derived from the REACTOME subset of canonical pathways in MSigDB are listed. The color of the dots represents the false discovery rate (FDR) value, and the diameter represents the enriched gene count.

it is not clear whether primary PDOs have high basal expression levels of these genes or whether they contain high numbers of stem-like and differentiated-like cells. To distinguish between these possibilities, we carried out single-cell RNA sequencing (scRNA-seq) analysis. PDOs established from patient HCT25 were chosen because the bulk gene expression analysis indicated that primary (HCT25-1T), metastasis (HCT25-5 LM), and recurrence (HCT25-10LMRR) results correlated well with those derived from the same tumor sites (Figures 1E and S3F). Multiple-dimensional scaling analysis of the combined data of these three PDOs revealed five major clusters, C1–C5 (Figure 2A) (Haber et al., 2017; Yan et al., 2017). Hierarchical clustering indicated that a number of proliferation markers, including PCNA, CDK1, and AURKA, were highly expressed in C2–C4 cells (Figure 2B). In contrast, differentiation markers, such as KRT19 and 20, were expressed in C5 and to a lesser extent in C4. These observations indicate that C2 and C3 are composed of actively proliferating cells but that C4 and C5 comprise progenitor-like and differentiated-like cells, respectively. These notions are supported by the close examination of differentially expressed genes (Figures 2C; Table S6): C2 and C3 highly expressed proliferation markers, including UBE2C (log fold change [logFC] = 1.46, p = 5.44E−100) and TOP2A (logFC = 1.13, p = 4.50E−51); whereas C4 and C5 expressed differentiation markers, such as TFF3 (logFC = 1.45, p = 2.23E−43), KRT20 (logFC = 1.23, p = 3.985E−26), and FABP1 (logFC = 0.92, p = 9.24E−19). Notably, OLFM4 was identified as the gene most significantly expressed in C1 (logFC = 0.81, p = 6.35E−15). In addition, we found that five ISC signature genes were highly expressed in C1: MYC (logFC = 0.54, p = 3.12E−13), IFITM3 (logFC = 0.45, p = E−10), KRT23 (logFC = 0.54, p = 3.12E−13), SMOC2 (logFC = 0.37, p = 1.11E−4), and CD44 (logFC = 0.28, p = 2.14E−4). Notably, none of these genes were positively related to other clusters, and CD44 was negatively related to C2, and MYC and OLFM4 were negatively related to C4. These observations suggest that C1 is enriched with stem-like cells. Consistent with these observations, detailed analysis of each marker gene revealed that OLFM4 and MYC were most highly expressed in C1 (Figures 2D and 2E). Two proliferation markers, UBE2C and TOP2A, were highly expressed in C2–C4 cells, and two differentiation markers, TFF3 and FABP1, were highly expressed in C4 and C5 cells.

To clarify the biological properties of each cluster, we performed GSEA using the Molecular Signature Database (MSigDB) (Figure 2F) (Subramanian et al., 2005). As expected, gene sets related to the cell cycle and mitosis were enriched in the cells in clusters C2, C3, and C4. Interestingly, gene sets related to canonical pathways derived from the metabolism of RNA, translation and eukaryotic translation initiation, which are upregulated in stem-like cells in various tumors (Blanco et al., 2016; Liakath-Ali et al., 2018; Signer et al., 2014), were enriched in the cells in cluster C1. In addition, RHO signal-related gene sets were identified as negatively correlating gene sets in the cells in cluster C1. These observations support the notion that cluster C1 contains stem-like cells (Koslow et al., 2019; Ohata et al., 2012).

Based on these observations, we conclude that PDOs are composed of one stem-like cell cluster (C1), two highly proliferating cell clusters (C2 and C3), one progenitor-like cell cluster (C4), and one differentiated-like cell cluster (C5). These observations demonstrate that the clusters of cellular heterogeneity in advanced CRC resemble those in normal tissues (Haber et al., 2017).

Different Cellular Heterogeneity between Primary and Metastatic PDOs

We next addressed whether the different expression profiles between primary and metastatic/recurrent PDOs are due to distinct cellular heterogeneity. According to a violin plot, HCT25-5LM and HCT25-10LMRR contained a reduced number of OLFM4^{+} cells compared with HCT25-1T (Figure 3A). The number of MYC^{+} cells was also lower in metastatic and recurrent PDOs than in primary PDOs. Similarly, cells expressing differentiation markers, including TFF3 and FABP1, were present at low levels in HCT25-5LM and -10LMRR. Conversely, larger numbers of proliferating cells were detected in metastatic and recurrent PDOs. These profiles were observed not only by analysis of individual marker genes but also by cellular cluster analysis. Indeed, cluster enrichment analysis demonstrated that HCT25-1T contained a greater population of cells that belong to clusters C1, C4, and C5 than HCT25-5LM and -10LMRR. Conversely, HCT25-5LM and -10LMRR contained larger populations of cells that belong to cluster C3 (Figures 3B and 3C). These analyses show that the profile of bulk gene expression analysis is due to the distinct cellular composition among lesions and that metastatic/recurrent PDOs contain fewer stem-like cell and differentiated-like cell clusters and more proliferating cell clusters.

Expression of OLFM4 in PDOs and Corresponding Surgical Specimens

To validate the transcriptome analyses, immunohistochemistry of OLFM4 was performed in PDOs and corresponding surgical specimens. We analyzed four sets of PDOs and the corresponding surgical specimens. HCT25-1T contained a greater number of OLFM4^{+} cells than HCT25-5LM and -10LMRR (Figures 4A and S4A). A reduced number of OLFM4^{+} cells in metastatic/recurrent PDOs was also observed in HCT41, -45, and −59.

H&E staining of the corresponding surgical specimens did not identify clear and distinct histopathological

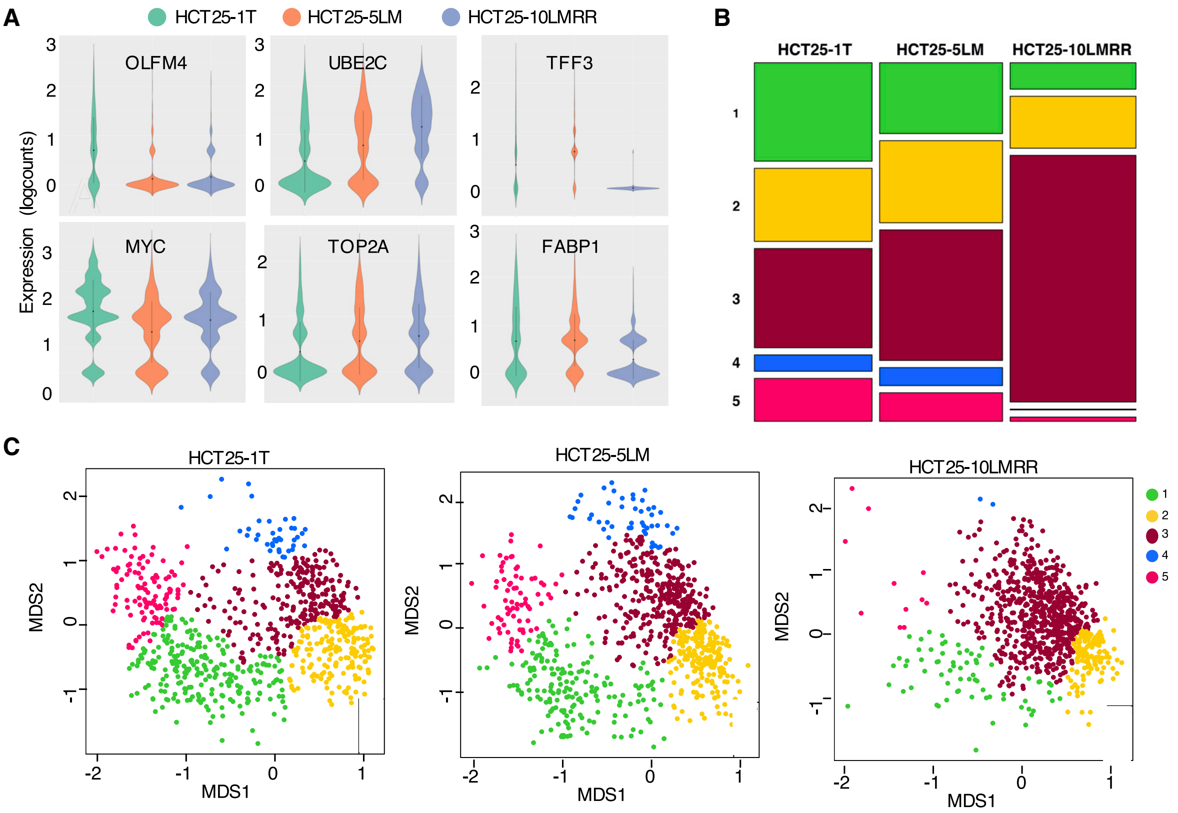

Figure 3. Differences in Expression of Marker Genes and Cellular Clusters between Primary and Metastatic PDOs

(A) Violin plot showing marker gene expression across PDOs. Distribution of expression of marker genes in the indicated PDOs. Expression is shown as the log of the total number of reads per cell (logcount).

(B) Proportions of clusters in each PDO. Cell-type clusters were determined by MDS as described in Figure 2A, and the proportions of each cluster in PDOs are shown.

(C) Cell-type clusters. MDS was used to visualize clustering based on unbiased gene expression analysis. Each cluster is colored.

features among the lesions (Figure 4B, upper panels). Nevertheless, immunohistochemistry identified topologically clustered OLFM4$^+$ cells in primary tumors, whereas thet were rarely detected in metastatic or recurrent lesions (Figures 4B, lower panels, S4B, and S4C). These observations indicate that the reduced expression of OLFM4 in metastatic and recurrent PDOs reflects the expression profile of tumor specimens.

OLFM4$^+$ Cells Are Indispensable for Efficient Reconstitution of Primary PDOs

OLFM4 has been shown to be a robust marker for stem cells in the human intestine (van der Flier et al., 2009a), but the biological roles of OLFM4$^+$ cells remain elusive. To evaluate the stemness of OLFM4$^+$ cells, an IRES-EGFP-P2A-iCaspase9 cassette was integrated into the 3′ UTR of the OLFM4 gene (Figure S5A). The donor cassette and sgRNA were introduced into HCT25-1T and -10LMRR cells, and correctly integrated clones were identified by Southern blot analysis (Figure S5B). Expression of EGFP in OLFM4$^+$ cells was validated by fluorescence-activated cell sorting (FACS) followed by RT-PCR (Figure S5C), and the expression of OLFM4 was 5.6 times higher in the EGFP$^+$ fraction than in the EGFP$^-$ fraction (Figure S5D). Specific expression of EGFP protein in OLFM4$^+$ cells was confirmed by immunofluorescence staining (Figure S5E). As expected, AP20187, which dimerizes and activates iCaspase9, rapidly ablated EGFP$^+$ cells (Figure S5F).

OLFM4$^+$ cells were obtained by fluorescence sorting, and the ability to regenerate the organoid was evaluated (Figure 5A). OLFM4$^+$ cells produced organoids 6.4 times more efficiently than OLFM4$^-$ cells ($p < 0.01$, unpaired t test) (Figures 5B and 5C). Similar observations were made in two additional PDO sets (Figures S5B, S6A, and S6B). Close examination of the reconstruction process revealed that the number of OLFM4$^+$ cells had multiplied during the first 3 days after plating, indicating their replication capability (Figure 5D). At 6 days, OLFM4$^-$ cells were evident. Immunofluorescence analysis indicated the presence of KRT20-expressing cells, showing the differentiation ability of

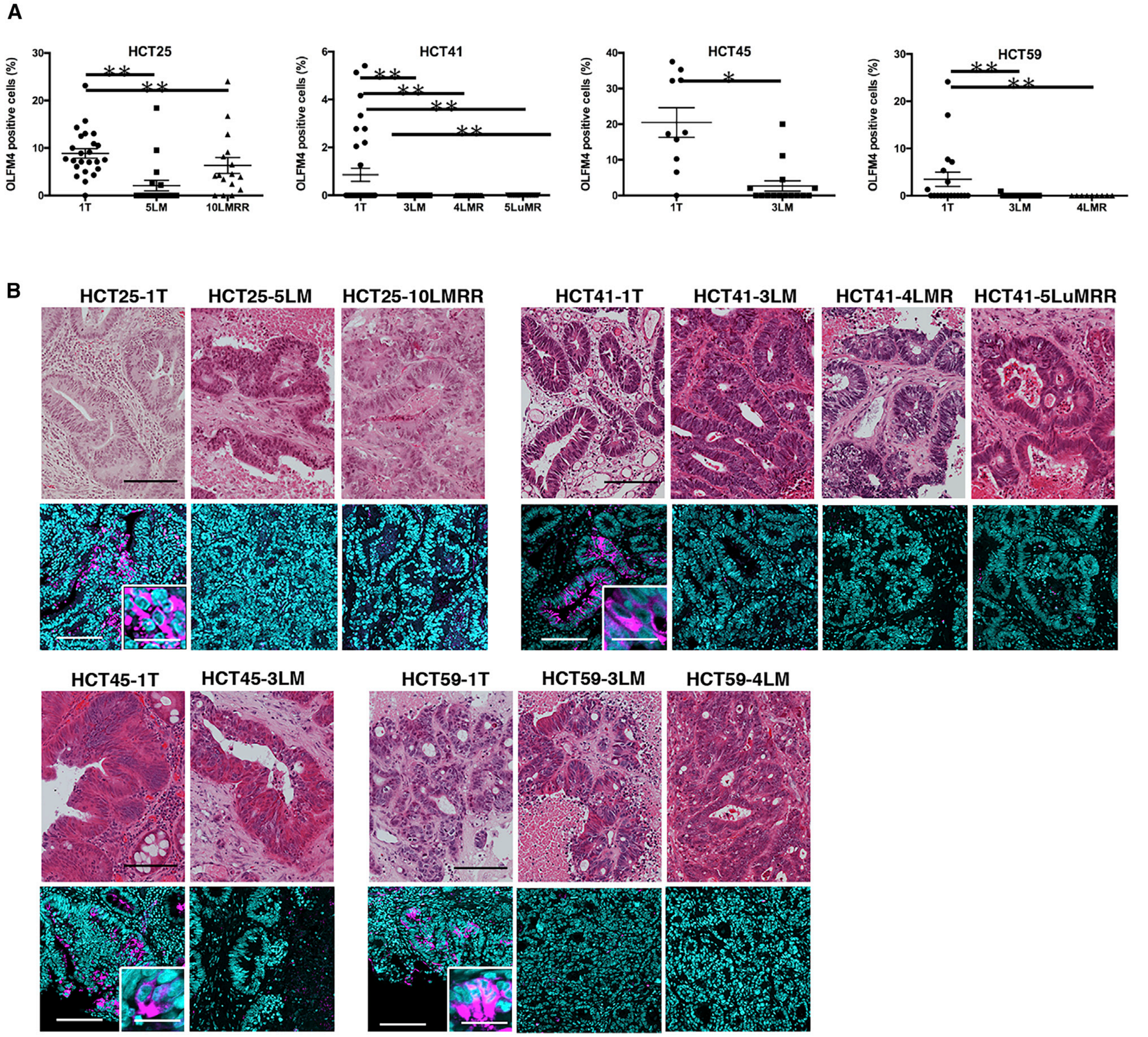

Figure 4. Immunohistochemical Analysis of OLFM4

(A) Quantification of $OLFM4^+$ cells in PDOs. Formalin fixed paraffin embedded (FFPE) sections of the indicated PDOs were probed with an anti-OLFM4 antibody. Nuclei were stained with DAPI. The number of $OLFM4^+$ cells and the total number of cells in each PDO were counted using a cell counter implemented in ImageJ. Each data point represents the percentage of $OLFM4^+$ cells per total number of cells. Data are shown as the mean and SD (n = 4 independent experiments). $^{**}p < 0.01$, $^{*}p < 0.05$ (unpaired t test). See also Figure S4.

(B) H&E images of FFPE sections of surgical specimens (upper panels). Scale bars, 100 μm. Immunofluorescence analysis using an anti-OLFM4 antibody (shown in magenta, lower panels) of surgical specimens from which the indicated PDOs were established. Nuclei were stained with DAPI (shown in cyan). Scale bars, 100 μm. Higher magnifications of $OLFM4^+$e cells are shown in the insets. Scale bars, 10 μm. See also Figure S4.

$OLFM4^+$ cells (Figure 5E). These observations demonstrate that $OLFM4^+$ cells are capable of initiating organoid culture growth.

Despite low efficiency, $OLFM4^-$ HCT25-1T cells were able to generate organoids (Figure 5C). We examined the reconstitution process and found that expression of EGFP was rapidly restored after the plating of single cells and that the organoid structure was formed (Figure 6A). Notably, exposure to AP20187 strongly interfered with the growth of organoids ($p < 0.01$, unpaired t test) (Figures

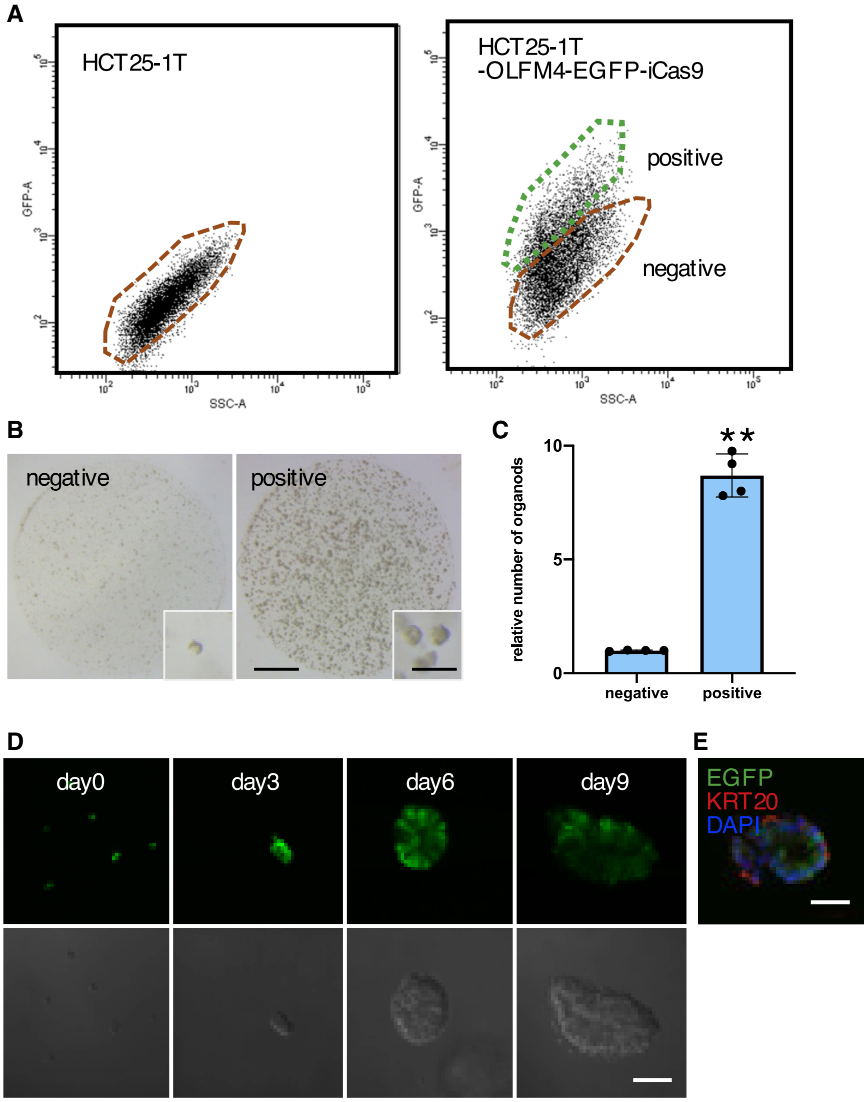

Figure 5. Organoid-Initiating Capacity and Differentiation Potential of OLFM4^{+} Cells in Primary PDOs

(A) FACS chart of HCT25-1T and HCT25-1T harboring the IRES-EGFP-P2A-iCaspase9 cassette in the 3′ UTR of the OLFM4 locus (HCT25-1T-OLFM4-EGFP-iCas9). The gates for EGFP^{+} and EGFP^{-} cells are shown in green and red, respectively.

(B) Reconstruction of organoids. Flow-sorted single HCT25-1T-OLFM4-EGFP-iCas9 cells (10,000 cells) were cultured for 9 days and analyzed under a stereomicroscope. Scale bars, 1,000 and 200 μm (inset).

(C) Organoid reconstruction efficiency from single cells. Flow-sorted single HCT25-1T-OLFM4-iCas9 cells were cultured as described in (B), and the number of organoids was counted. The number of organoids relative to that of organoids generated from OLFM4^{-} cells is shown. Data are shown as the mean and SD. $**p < 0.01$ (unpaired t test, n = 4 independent experiments).

(D) Expression of EGFP during reconstitution. Flow-sorted HCT25-1T-OLFM4-iCas9 cells were cultured for the indicated days and analyzed under a confocal microscope. Scale bar, 50 μm.

(E) Expression of KRT20. Organoids were recovered 7 days after starting a single-cell culture and stained with an anti-KRT20 antibody. Nuclei were visualized using DAPI. Scale bar, 50 μm.

See also Figures S5 and S6.

6B and 6C). AP20187 also suppressed the generation of organoids from OLFM4^{-} cells in HCT26-1T and HCT41-1T cells ($p < 0.01$, unpaired t test) (Figure S6C). Although the effects of AP20187 varied depending on the line, these observations indicate that a fraction of OLFM4^{-} cells possess the potential to revert to OLFM4^{+} cells, which is indispensable for the efficient growth of PDOs.

Metastatic/recurrent PDOs Do Not Require OLFM4^{+} Cells for the Formation of Organoids

We next assessed the role of OLFM4^{+} cells in the organoid formation of metastatic PDOs. Consistent with the transcriptome and immunofluorescence analyses, a lower number of EGFP^{+} cells was found for HCT25-10LMRR than HCT25-1T (Figure 7A). Notably, OLFM4^{-} cells efficiently generated organoids and, in contrast to the HCT25-1T OLFM4^{-} cells, AP20187 treatment did not interfere with their growth (Figures 7B and 7C). Furthermore, OLFM4 was not expressed during organoid formation (Figure 7D). Similar observations were made in a PDO derived from the metastatic lesion HCT26-3LM (Figure S7). Thus, it appears that a subset of metastatic/recurrent PDOs did not depend on OLFM4^{+} cells for their efficient growth. These observations reveal functionally different control of cellular heterogeneity between primary PDOs and patient-matched metastatic PDOs.

DISCUSSION

We established PDOs derived from patient-matched primary and metastatic/recurrent tumors. Biobanks of PDOs established from various cancer types have been described

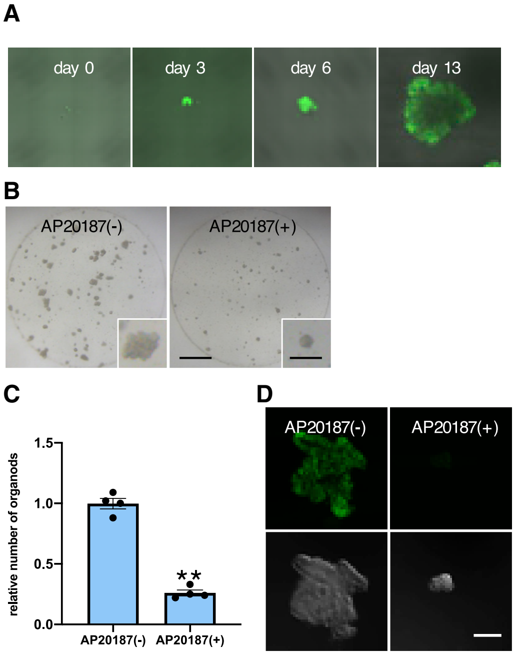

Figure 6. Reconstruction of Organoids from OLFM4^{-} Cells in Primary PDOs

(A) Re-expression of EGFP in EGFP^{-} cells. HCT25-1T-OLFM4_EGFP-iCas9 was dissociated into single cells, and FACS-sorted EGFP^{-} cells were cultured for the indicated days. Expression of EGFP was analyzed using confocal microscopy.

(B) AP20187 suppressed the growth of EGFP^{-} HCT25-1T-OLFM4_EGFP-iCas9 cells. Flow-sorted EGFP^{-} cells (10,000 cells) were cultured with or without AP20187 for 14 days. Scale bars, 1,000 and 200 μm (inset).

(C) Efficiency of organoid reconstitution. Flow-sorted EGFP^{-} HCT25-1T-OLFM4_EGFP-iCas9 cells were cultured as described in (B). The number of organoids was counted, and the result is shown as the relative number of organoids generated without AP20187. Data are shown as the mean and SD. $^{**}p < 0.01$ (unpaired t test, n = 4 independent experiments).

(D) Growth suppression of organoids by AP20187. EGFP^{-} cells were isolated and cultured with or without AP20187 for 9 days. Organoid structure and EGFP expression were analyzed using confocal microscopy.

See also Figures S5 and S6.

(Drost and Clevers, 2018; Tuveson and Clevers, 2019), including CRC (Fujii et al., 2016; van de Wetering et al., 2015). A growing body of research shows that PDOs represent the original tumors, including genetic mutations, histopathological features, and the response to chemotherapeutic agents (Engel et al., 2020; Johnson et al., 2020; Ooft et al., 2019; Vlachogiannis et al., 2018; Weeber et al., 2015). In this study, we focused on metastases and established 72 PDOs from 21 stage IV CRC patients by collecting synchronous metastatic tumors as well as recurrent lesions by following up with patients after surgery for the primary tumor. These patient-matched PDO sets exhibited identical genetic backgrounds and shared the cells of origin, allowing us to explore the altered profiles during the metastatic process and to evaluate their biological significance.

Intratumor heterogeneity is a key issue in understanding human cancer. In general, previous histopathological and transcriptome analyses support the notion that tumor tissues contain multiple cell types and that their organization resembles that of normal tissues. To identify the marker genes in human tumors in an unbiased way, we explored the cellular heterogeneity of PDOs derived from CRC surgical specimens and searched for marker genes. scRNA-seq analysis of a set of PDOs derived from one patient indicated that these organoids consisted of at least five clusters. In addition, differentially expressed gene analysis DEG analysis revealed six ISC signature genes to be highly expressed in cluster C1 (Figure 2C). GSEA using MSigDB showed that gene sets related to RNA processing and translation were upregulated but that Rho GTPase effectors were downregulated (Figure 2F). These observations suggest that cluster C1 was enriched with stem-like cells. We identified OLFM4 as the gene most significantly associated with cluster C1. scRNA-seq analysis was performed on only one set of PDOs derived from one patient, but OLFM4 expression in primary tumors was validated by immunofluorescence analysis of four sets of PDOs and their corresponding surgical specimens (Figures 4 and S4). Biologically, we showed that single OLFM4^{+} cells have organoid-initiating and differentiation capacity and can reconstitute the organoid structure. These observations demonstrate that OLFM4^{+} cells are functionally stem-like cells in human CRC.

In this study, we explored the difference between primary and metastatic CRC and identified OLFM4 as the most differentially expressed gene in a comparative expression analysis between primary PDOs and patient-matched metastatic PDOs. Nonetheless, previous transcriptome analysis of surgical specimens revealed largely similar expression profiles between primary tumors and metastatic lesions (Lee et al., 2016; Vignot et al., 2015). This discrepancy is most likely due to the nontumor cells present in the tumor specimen, which cannot grow under organoid culture conditions. The cellular composition of tumor tissues is controlled by mesenchymal cells, including cancer associated fibroblasts and immune cells. Recent studies have reported that stem cell functionality is defined by the microenvironment (Lenos et al., 2018; Vermeulen et al., 2010). Because the primary and metastatic/recurrent PDOs were cultured in identical medium, our findings showing different expression of OLFM4 represent the intrinsic properties of tumor cells.

Overall, the biological roles of OLFM4^{+} cells in metastasis remain to be elucidated. In the mouse model of metastasis, Lgr5^{+} cells were shown to act as cancer-initiating cells and

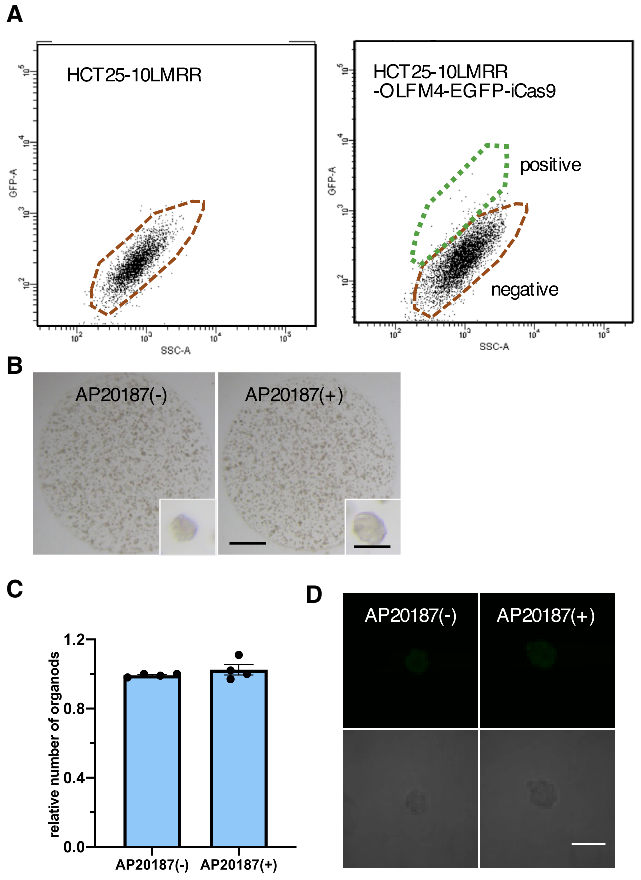

Figure 7. Generation of Organoids from OLFM4^{-} Single Cells in Metastatic PDOs

(A) FACS chart of HCT25-10LMRR and HCT25-10LMRR harboring the IRES-EGFP-P2A-iCaspase9 cassette in the 3′ UTR of the OLFM4 locus (HCT25-10LMRR-OLFM4-EGFP-iCas9). The gates for EGFP^{+} and EGFP^{-} cells are shown in green and red, respectively.

(B) AP20187 did not suppress the growth of EGFP^{-} HCT25-10LMRR-OLFM4_EGFP-iCas9 cells. Flow-sorted EGFP^{-} cells (10,000 cells) were cultured with or without AP20187 for 9 days. Scale bars, 1,000 and 200 μm (inset).

(C) Efficiency of organoid reconstitution. Flow-sorted EGFP^{-} HCT25-10LMRR-OLFM4-iCas9 cells were cultured as described in (B). The number of organoids was counted, and the result is shown as the relative number of organoids generated without AP20187. Data are shown as the mean and SD. **p < 0.01 (unpaired t test, n = 4 independent experiments).

(D) Lack of EGFP expression in metastatic PDOs. Flow-sorted EGFP^{-} HCT25-10LMRR-OLFM4-iCas9 cells were cultured for 7 days and analyzed under a confocal microscope. Scale bar, 50 μm.

See also Figures S5 and S7.

to be essential for metastasis (de Sousa e Melo et al., 2017). A more recent study reported that most disseminated cells in the circulation were Lgr5^{-}, forming distant metastases where Lgr5^{+} occurred (Fumagalli et al., 2020). These observations indicate that the stemness and cellular plasticity of Lgr5^{+} cells play critical roles in the metastasis process. In this study, we showed that OLFM4^{+} cells were capable of initiating organoid culture growth and displayed differentiation capacity in primary PDOs. We also showed that a subset of OLFM4^{-} cells can produce OLFM4^{+} cells. These observations suggest their stem cell-like properties and cellular plasticity. To examine the roles of OLFM4^{+} cells in the metastasis process, it might be useful to develop a mouse model of metastasis by transplanting PDOs into the colon mucosa.

Previous scRNA-seq profiling of normal tissues and tumors has revealed a dramatic increase in stem/transient amplifying-like cells and a decrease in differentiated-like cells (Dalerba et al., 2011; Li et al., 2017). Reduced expression of marker genes for differentiated-like cells was observed in our scRNA-seq analysis. The repeated-measures bulk transcriptome analysis of our cohort identified differentiated cell markers as being significantly decreased among genes expressed in metastatic/recurrent PDOs. These observations show that, at least in a subset of PDO sets, metastatic/recurrent PDOs possess less variable cellular hierarchy than primary PDOs. These differences in cellular composition are the potential cause of the divergent response to chemotherapy and/or distinct prognosis among stage IV CRC patients because cellular heterogeneity is considered to be a key factor contributing to resistance to chemotherapeutic agents. Further comprehensive analysis of patient-matched primary and metastatic/recurrent PDOs may provide a clue for developing novel therapeutic strategies for advanced CRC.

EXPERIMENTAL PROCEDURES

For details of this section, please also refer to the Supplemental Experimental Procedures.

In brief, PDOs were established and cultured in Advanced DMEM/F12 (Gibco) supplemented with 10 ng/mL EGF (Invitrogen), 10% Noggin conditioned medium, and 1 μg/ml R-spondin-1 (R&D Systems) at 37°C in 5% O_2 as described previously (Sakahara et al., 2019). Targetory sequencing was performed using a MiSeq system (Illumina) as described (Sakahara et al., 2019). Expression profile was obtained using Human Transcriptome Array 2 (Affymetrix) and data were analyzed using Transcriptome Analysis Console (Affymetrix). scRNA-seq analysis was performed using the Chromium system (10x Genomics) and the libraries were sequenced using HiSeq 2500 system (Illumina).

We used CRISPR-Cas9-mediated homology-independent targeted integration to insert the IRES-EGFP-P2A-iCaspase9 cassette into 3′ UTR of OLFM4 (Suzuki et al., 2016). For FACS analysis, PDOs were dissociated using TripleLE Express (Life Technologies), and the cells were sorted using a 100 μm nozzle (Aria III, BD Biosciences). Cells (1×10^4) were embedded in 25 μL of Matrigel in a 48-well plate and images were analyzed using the cell counter plugin installed in ImageJ (v.2.0.0). Data are presented as the mean and SD (error bars) of four independent experiments.

Data and Code Availability

The microarray data have been deposited in the Gene Expression Omnibus under accession number GSE128213. The scRNA-seq data have been deposited in the DDBJ Japanese Genotype-phenotype Archive (JGA) for genetic and phenotypic human data database under accession code JGAS00000000139.

SUPPLEMENTAL INFORMATION

Supplemental Information can be found online at https://doi.org/10.1016/j.stemcr.2021.02.012.

AUTHOR CONTRIBUTIONS

R.Y. and S.N. planned the study, established the PDOs, and prepared the manuscript. T.N., Y. Sakai, M.U., and Y.T. supervised the project. T.O., K.Y., and R.Y. contributed to the gene array analysis, cell culture, and transplantation experiments. Y.N., D.K., and M.Y. contributed to the establishment and culture of the PDOs, and H.Y. and M.F. contributed to the screening of the genome-edited PDOs. Y. Kashima, Y. Suzuki, A.S., and Y. Kuze performed the scRNA-seq experiments and data analysis. D.d.V., F.P., U.S., and K.T. performed the bioinformatics analysis.

CONFLICTS OF INTEREST

The authors declare that they have no conflicts of interest.

ACKNOWLEDGMENTS

We thank Y. Ota (Japanese Foundation for Cancer Research) for performing the microarray experiments. This work was supported in part by MEXT/JSPS KAKENHI grant nos. 16H06276 (to R.Y.), 16K14620 (to R.Y.), 17H06333 (to R.Y.), 18H02684 (to R.Y.), and 16H06279 (PAGS), and by JST CREST grant no. JPMJCR1502 (to K.T.).

Received: March 19, 2020
Revised: February 11, 2021
Accepted: February 12, 2021
Published: March 11, 2021

REFERENCES

Barker, N. (2014). Adult intestinal stem cells: critical drivers of epithelial homeostasis and regeneration. Nat. Rev. Mol. Cell Biol. *15*, 19–33.

Barker, N., van Es, J.H., Kuipers, J., Kujala, P., van den Born, M., Cozijnsen, M., Haegebarth, A., Korving, J., Begthel, H., Peters, P.J., et al. (2007). Identification of stem cells in small intestine and colon by marker gene Lgr5. Nature *449*, 1003–1007.

Bedard, P.L., Hansen, A.R., Ratain, M.J., and Siu, L.L. (2013). Tumour heterogeneity in the clinic. Nature *501*, 355–364.

Birkbak, N.J., and McGranahan, N. (2020). Cancer genome evolutionary trajectories in metastasis. Cancer Cell *37*, 8–19.

Blanco, S., Bandiera, R., Popis, M., Hussain, S., Lombard, P., Aleksic, J., Sajini, A., Tanna, H., Cortes-Garrido, R., Gkatza, N., et al. (2016). Stem cell function and stress response are controlled by protein synthesis. Nature *534*, 335–340.

Boj, S.F., Hwang, C.I., Baker, L.A., Chio, I.I., Engle, D.D., Corbo, V., Jager, M., Ponz-Sarvise, M., Tiriac, H., Spector, M.S., et al. (2015). Organoid models of human and mouse ductal pancreatic cancer. Cell *160*, 324–338.

Clevers, H. (2013). The intestinal crypt, a prototype stem cell compartment. Cell *154*, 274–284.

Cortina, C., Turon, G., Stork, D., Hernando-Momblona, X., Sevillano, M., Aguilera, M., Tosi, S., Merlos-Suarez, A., Stephan-Otto Attolini, C., Sancho, E., et al. (2017). A genome editing approach to study cancer stem cells in human tumors. EMBO Mol. Med. *9*, 869–879.

Dalerba, P., Kalisky, T., Sahoo, D., Rajendran, P.S., Rothenberg, M.E., Leyrat, A.A., Sim, S., Okamoto, J., Johnston, D.M., Qian, D., et al. (2011). Single-cell dissection of transcriptional heterogeneity in human colon tumors. Nat. Biotechnol. *29*, 1120–1127.

de Sousa e Melo, F., Kurtova, A.V., Harnoss, J.M., Kljavin, N., Hoeck, J.D., Hung, J., Anderson, J.E., Storm, E.E., Modrusan, Z., Koeppen, H., et al. (2017). A distinct role for Lgr5(+) stem cells in primary and metastatic colon cancer. Nature *543*, 676–680.

Drost, J., and Clevers, H. (2018). Organoids in cancer research. Nat. Rev. Cancer *18*, 407–418.

Eide, P.W., Bruun, J., Lothe, R.A., and Sveen, A. (2017). CMScaller: an R package for consensus molecular subtyping of colorectal cancer pre-clinical models. Sci. Rep. *7*, 16618.

Engel, R.M., Chan, W.H., Nickless, D., Hlavca, S., R ichards, E., Kerr, G., Oliva, K., McMurrick, P.J., Jarde, T., and Abud, H.E. (2020). Patient-derived colorectal cancer organoids upregulate revival stem cell marker genes following chemotherapeutic treatment. J. Clin. Med. *9*, 128.

Fujii, M., Shimokawa, M., Date, S., Takano, A., Matano, M., Nanki, K., Ohta, Y., Toshimitsu, K., Nakazato, Y., Kawasaki, K., et al. (2016). A colorectal tumor organoid library demonstrates progressive loss of niche factor requirements during tumorigenesis. Cell Stem Cell *18*, 827–838.

Fumagalli, A., Oost, K.C., Kester, L., Morgner, J., Bornes, L., Bruens, L., Spaargaren, L., Azkanaz, M., Schelfhorst, T., Beerling, E., et al. (2020). Plasticity of Lgr5-negative cancer cells drives metastasis in colorectal cancer. Cell Stem Cell *26*, 569–578.e7.

Guinney, J., Dienstmann, R., Wang, X., de Reynies, A., Schlicker, A., Soneson, C., Marisa, L., Roepman, P., Nyamundanda, G., Angelino, P., et al. (2015). The consensus molecular subtypes of colorectal cancer. Nat. Med. *21*, 1350–1356.

Haber, A.L., Biton, M., Rogel, N., Herbst, R.H., Shekhar, K., Smillie, C., Burgin, G., Delorey, T.M., Howitt, M.R., Katz, Y., et al. (2017). A single-cell survey of the small intestinal epithelium. Nature *551*, 333–339.

Huang, L., Holtzinger, A., Jagan, I., BeGora, M., Lohse, I., Ngai, N., Nostro, C., Wang, R., Muthuswamy, L.B., Crawford, H.C., et al. (2015). Ductal pancreatic cancer modeling and drug screening using human pluripotent stem cell- and patient-derived tumor organoids. Nat. Med. *21*, 1364–1371.

Ikehara, Y., Kojima, N., Kurosawa, N., Kudo, T., Kono, M., Nishihara, S., Issiki, S., Morozumi, K., Itzkowitz, S., Tsuda, T., et al. (1999). Cloning and expression of a human gene encoding an N-acetylgalactosamine-alpha2,6-sialyltransferase (ST6GalNAc I): a

candidate for synthesis of cancer-associated sialyl-Tn antigens. Glycobiology *9*, 1213–1224.

Ishaque, N., Abba, M.L., Hauser, C., Patil, N., Paramasivam, N., Huebschmann, D., Leupold, J.H., Balasubramanian, G.P., Kleinheinz, K., Toprak, U.H., et al. (2018). Whole genome sequencing puts forward hypotheses on metastasis evolution and therapy in colorectal cancer. Nat. Commun. *9*, 4782.

Iusuf, D., Teunissen, S.F., Wagenaar, E., Rosing, H., Beijnen, J.H., and Schinkel, A.H. (2011). P-Glycoprotein (ABCB1) transports the primary active tamoxifen metabolites endoxifen and 4-hydroxytamoxifen and restricts their brain penetration. J. Pharmacol. Exp. Ther. *337*, 710–717.

Johnson, K.A., DeStefanis, R.A., Emmerich, P.B., Grogan, P.T., Kratz, J.D., Makkar, S.K., Clipson, L., and Deming, D.A. (2020). Human colon organoids and other laboratory strategies to enhance patient treatment selection. Curr. Treat. Options Oncol. *21*, 35.

Karthaus, W.R., Iaquinta, P.J., Drost, J., Gracanin, A., van Boxtel, R., Wongvipat, J., Dowling, C.M., Gao, D., Begthel, H., Sachs, N., et al. (2014). Identification of multipotent luminal progenitor cells in human prostate organoid cultures. Cell *159*, 163–175.

Kiyotani, K., Mushiroda, T., Imamura, C.K., Hosono, N., Tsunoda, T., Kubo, M., Tanigawara, Y., Flockhart, D.A., Desta, Z., Skaar, T.C., et al. (2010). Significant effect of polymorphisms in CYP2D6 and ABCC2 on clinical outcomes of adjuvant tamoxifen therapy for breast cancer patients. J. Clin. Oncol. *28*, 1287–1293.

Koslow, M., O'Keefe, K.J., Hosseini, Z.F., Nelson, D.A., and Larsen, M. (2019). ROCK inhibitor increases proacinar cells in adult salivary gland organoids. Stem Cell Res *41*, 101608.

Lambert, A.W., Pattabiraman, D.R., and Weinberg, R.A. (2017). Emerging biological principles of metastasis. Cell *168*, 670–691.

Lee, J.R., Kwon, C.H., Choi, Y., Park, H.J., Kim, H.S., Jo, H.J., Oh, N., and Park do, Y. (2016). Transcriptome analysis of paired primary colorectal carcinoma and liver metastases reveals fusion transcripts and similar gene expression profiles in primary carcinoma and liver metastases. BMC Cancer *16*, 539.

Lenos, K.J., Miedema, D.M., Lodestijn, S.C., Nijman, L.E., van den Bosch, T., Romero Ros, X., Lourenco, F.C., Lecca, M.C., van der Heijden, M., van Neerven, S.M., et al. (2018). Stem cell functionality is microenvironmentally defined during tumour expansion and therapy response in colon cancer. Nat. Cell Biol. *20*, 1193–1202.

Li, H., Courtois, E.T., Sengupta, D., Tan, Y., Chen, K.H., Goh, J.J.L., Kong, S.L., Chua, C., Hon, L.K., Tan, W.S., et al. (2017). Reference component analysis of single-cell transcriptomes elucidates cellular heterogeneity in human colorectal tumors. Nat. Genet. *49*, 708–718.

Liakath-Ali, K., Mills, E.W., Sequeira, I., Lichtenberger, B.M., Pisco, A.O., Sipila, K.H., Mishra, A., Yoshikawa, H., Wu, C.C., Ly, T., et al. (2018). An evolutionarily conserved ribosome-rescue pathway maintains epidermal homeostasis. Nature *556*, 376–380.

Magee, J.A., Piskounova, E., and Morrison, S.J. (2012). Cancer stem cells: impact, heterogeneity, and uncertainty. Cancer Cell *21*, 283–296.

Massague, J., and Obenauf, A.C. (2016). Metastatic colonization by circulating tumour cells. Nature *529*, 298–306.

Meacham, C.E., and Morrison, S.J. (2013). Tumour heterogeneity and cancer cell plasticity. Nature *501*, 328–337.

Munoz, J., Stange, D.E., Schepers, A.G., van de Wetering, M., Koo, B.K., Itzkovitz, S., Volckmann, R., Kung, K.S., Koster, J., Radulescu, S., et al. (2012). The Lgr5 intestinal stem cell signature: robust expression of proposed quiescent '+4' cell markers. EMBO J. *31*, 3079–3091.

Nanki, K., Toshimitsu, K., Takano, A., Fujii, M., Shimokawa, M., Ohta, Y., Matano, M., Seino, T., Nishikori, S., Ishikawa, K., et al. (2018). Divergent routes toward Wnt and R-spondin niche independency during human gastric carcinogenesis. Cell *174*, 856–869 e817.

Ohata, H., Ishiguro, T., Aihara, Y., Sato, A., Sakai, H., Sekine, S., Taniguchi, H., Akasu, T., Fujita, S., Nakagama, H., et al. (2012). Induction of the stem-like cell regulator CD44 by Rho kinase inhibition contributes to the maintenance of colon cancer-initiating cells. Cancer Res. *72*, 5101–5110.

Ooft, S.N., Weeber, F., Dijkstra, K.K., McLean, C.M., Kaing, S., van Werkhoven, E., Schipper, L., Hoes, L., Vis, D.J., van de Haar, J., et al. (2019). Patient-derived organoids can predict response to chemotherapy in metastatic colorectal cancer patients. Sci. Translational Med. *11*, eaay2574.

Sachs, N., de Ligt, J., Kopper, O., Gogola, E., Bounova, G., Weeber, F., Balgobind, A.V., Wind, K., Gracanin, A., Begthel, H., et al. (2018). A living biobank of breast cancer organoids captures disease heterogeneity. Cell *172*, 373–386 e310.

Sakahara, M., Okamoto, T., Oyanagi, J., Takano, H., Natsume, Y., Yamanaka, H., Kusama, D., Fusejima, M., Tanaka, N., Mori, S., et al. (2019). IFN/STAT signaling controls tumorigenesis and the drug response in colorectal cancer. Cancer Sci. *110*, 1293–1305.

Sato, T., Vries, R.G., Snippert, H.J., van de Wetering, M., Barker, N., Stange, D.E., van Es, J.H., Abo, A., Kujala, P., Peters, P.J., et al. (2009). Single Lgr5 stem cells build crypt-villus structures in vitro without a mesenchymal niche. Nature *459*, 262–265.

Schepers, A.G., Snippert, H.J., Stange, D.E., van den Born, M., van Es, J.H., van de Wetering, M., and Clevers, H. (2012). Lineage tracing reveals Lgr5+ stem cell activity in mouse intestinal adenomas. Science *337*, 730–735.

Schlicker, A., Ellappalayam, A., Beumer, I.J., Snel, M.H.J., Mittempergher, L., Diosdado, B., Dreezen, C., Tian, S., Salazar, R., Loupakis, F., et al. (2020). Investigating the concordance in molecular subtypes of primary colorectal tumors and their matched synchronous liver metastasis. Int. J. Cancer *147*, 2303–2315.

Schweiger, T., Liebmann-Reindl, S., Glueck, O., Starlinger, P., Laengle, J., Birner, P., Klepetko, W., Pils, D., Streubel, B., and Hoetzenecker, K. (2018). Mutational profile of colorectal cancer lung metastases and paired primary tumors by targeted next generation sequencing: implications on clinical outcome after surgery. J. Thorac. Dis. *10*, 6147–6157.

Sensorn, I., Sirachainan, E., Chamnanphon, M., Pasomsub, E., Trachu, N., Supavilai, P., Sukasem, C., and Pinthong, D. (2013). Association of CYP3A4/5, ABCB1 and ABCC2 polymorphisms and clinical outcomes of Thai breast cancer patients treated with tamoxifen. Pharmgenomics Pers Med. *6*, 93–98.

Sensorn, I., Sukasem, C., Sirachainan, E., Chamnanphon, M., Pasomsub, E., Trachu, N., Supavilai, P., Pinthong, D., and Wongwaisayawan, S. (2016). ABCB1 and ABCC2 and the risk of distant metastasis in Thai breast cancer patients treated with tamoxifen. Onco Targets Ther. *9*, 2121–2129.

Shimokawa, M., Ohta, Y., Nishikori, S., Matano, M., Takano, A., Fujii, M., Date, S., Sugimoto, S., Kanai, T., and Sato, T. (2017). Visualization and targeting of LGR5(+) human colon cancer stem cells. Nature *545*, 187–192.

Signer, R.A., Magee, J.A., Salic, A., and Morrison, S.J. (2014). Haematopoietic stem cells require a highly regulated protein synthesis rate. Nature *509*, 49–54.

Subramanian, A., Tamayo, P., Mootha, V.K., Mukherjee, S., Ebert, B.L., Gillette, M.A., Paulovich, A., Pomeroy, S.L., Golub, T.R., Lander, E.S., et al. (2005). Gene set enrichment analysis: a knowledge-based approach for interpreting genome-wide expression profiles. Proc. Natl. Acad. Sci. U S A *102*, 15545–15550.

Suzuki, K., Tsunekawa, Y., Hernandez-Benitez, R., Wu, J., Zhu, J., Kim, E.J., Hatanaka, F., Yamamoto, M., Araoka, T., Li, Z., et al. (2016). In vivo genome editing via CRISPR/Cas9 mediated homology-independent targeted integration. Nature *540*, 144–149.

Taniguchi, K., Wada, M., Kohno, K., Nakamura, T., Kawabe, T., Kawakami, M., Kagotani, K., Okumura, K., Akiyama, S., and Kuwano, M. (1996). A human canalicular multispecific organic anion transporter (cMOAT) gene is overexpressed in cisplatin-resistant human cancer cell lines with decreased drug accumulation. Cancer Res. *56*, 4124–4129.

Tieng, F.Y.F., Baharudin, R., Abu, N., Mohd Yunos, R.I., Lee, L.H., and Ab Mutalib, N.S. (2020). Single cell transcriptome in colorectal cancer-current updates on its application in metastasis, chemoresistance and the roles of circulating tumor cells. Front. Pharmacol. *11*, 135.

Tuveson, D., and Clevers, H. (2019). Cancer modeling meets human organoid technology. Science *364*, 952–955.

van de Wetering, M., Francies, H.E., Francis, J.M., Bounova, G., Iorio, F., Pronk, A., van Houdt, W., van Gorp, J., Taylor-Weiner, A., Kester, L., et al. (2015). Prospective derivation of a living organoid biobank of colorectal cancer patients. Cell *161*, 933–945.

van der Flier, L.G., Haegebarth, A., Stange, D.E., van de Wetering, M., and Clevers, H. (2009a). OLFM4 is a robust marker for stem cells in human intestine and marks a subset of colorectal cancer cells. Gastroenterology *137*, 15–17.

van der Flier, L.G., van Gijn, M.E., Hatzis, P., Kujala, P., Haegebarth, A., Stange, D.E., Begthel, H., van den Born, M., Guryev, V., Oving, I., et al. (2009b). Transcription factor achaete scute-like 2 controls intestinal stem cell fate. Cell *136*, 903–912.

Vermeulen, L., De Sousa, E.M.F., van der Heijden, M., Cameron, K., de Jong, J.H., Borovski, T., Tuynman, J.B., Todaro, M., Merz, C., Rodermond, H., et al. (2010). Wnt activity defines colon cancer stem cells and is regulated by the microenvironment. Nat. Cell Biol. *12*, 468–476.

Vignot, S., Lefebvre, C., Frampton, G.M., Meurice, G., Yelensky, R., Palmer, G., Capron, F., Lazar, V., Hannoun, L., Miller, V.A., et al. (2015). Comparative analysis of primary tumour and matched metastases in colorectal cancer patients: evaluation of concordance between genomic and transcriptional profiles. Eur. J. Cancer *51*, 791–799.

Vlachogiannis, G., Hedayat, S., Vatsiou, A., Jamin, Y., Fernandez-Mateos, J., Khan, K., Lampis, A., Eason, K., Huntingford, I., Burke, R., et al. (2018). Patient-derived organoids model treatment response of metastatic gastrointestinal cancers. Science *359*, 920–926.

Weeber, F., van de Wetering, M., Hoogstraat, M., Dijkstra, K.K., Krijgsman, O., Kuilman, T., Gadellaa-van Hooijdonk, C.G., van der Velden, D.L., Peeper, D.S., Cuppen, E.P., et al. (2015). Preserved genetic diversity in organoids cultured from biopsies of human colorectal cancer metastases. Proc. Natl. Acad. Sci. U S A *112*, 13308–13311.

Xie, S.M., Fang, W.Y., Liu, T.F., Yao, K.T., and Zhong, X.Y. (2010). Association of ABCC2 and CDDP-resistance in two sublines resistant to CDDP derived from a human nasopharyngeal carcinoma cell line. J. Oncol. *2010*, 915046.

Xie, T., Cho, Y.B., Wang, K., Huang, D., Hong, H.K., Choi, Y.L., Ko, Y.H., Nam, D.H., Jin, J., Yang, H., et al. (2014). Patterns of somatic alterations between matched primary and metastatic colorectal tumors characterized by whole-genome sequencing. Genomics *104*, 234–241.

Yan, K.S., Janda, C.Y., Chang, J., Zheng, G.X.Y., Larkin, K.A., Luca, V.C., Chia, L.A., Mah, A.T., Han, A., Terry, J.M., et al. (2017). Non-equivalence of Wnt and R-spondin ligands during Lgr5(+) intestinal stem-cell self-renewal. Nature *545*, 238–242.

Yang, Q., Bermingham, N.A., Finegold, M.J., and Zoghbi, H.Y. (2001). Requirement of Math1 for secretory cell lineage commitment in the mouse intestine. Science *294*, 2155–2158.

Stem Cell Reports, Volume *16*

Supplemental Information

Comparative Analysis of Patient-Matched PDOs Revealed a Reduction in OLFM4-Associated Clusters in Metastatic Lesions in Colorectal Cancer

Takuya Okamoto, David duVerle, Katsuyuki Yaginuma, Yasuko Natsume, Hitomi Yamanaka, Daisuke Kusama, Mayuko Fukuda, Mayuko Yamamoto, Fanny Perraudeau, Upasna Srivastava, Yukie Kashima, Ayako Suzuki, Yuuta Kuze, Yu Takahashi, Masashi Ueno, Yoshiharu Sakai, Tetsuo Noda, Koji Tsuda, Yutaka Suzuki, Satoshi Nagayama, and Ryoji Yao

Supplemental Information

Comparative analysis of patient-matched PDOs revealed the reduction of OLFM4-associated clusters in metastatic lesions in colorectal cancer.

Takuya Okamoto, David duVerle, Katsuyuki Yaginuma, Yasuko Natsume, Hitomi Yamanaka, Daisuke Kusama, Mayuko Fukuda, Mayuko Yamamoto, Fanny Perraudeau, Upasna Srivastava, Yukie Kashima, Ayako Suzuki, Yuuta Kuze, Yu Takahashi, Masashi Ueno, Yoshiharu Sakai, Tetsuo Noda, Koji Tsuda, Yutaka Suzuki, Satoshi, Nagayama, Ryoji Yao

Supplementary Figure 1

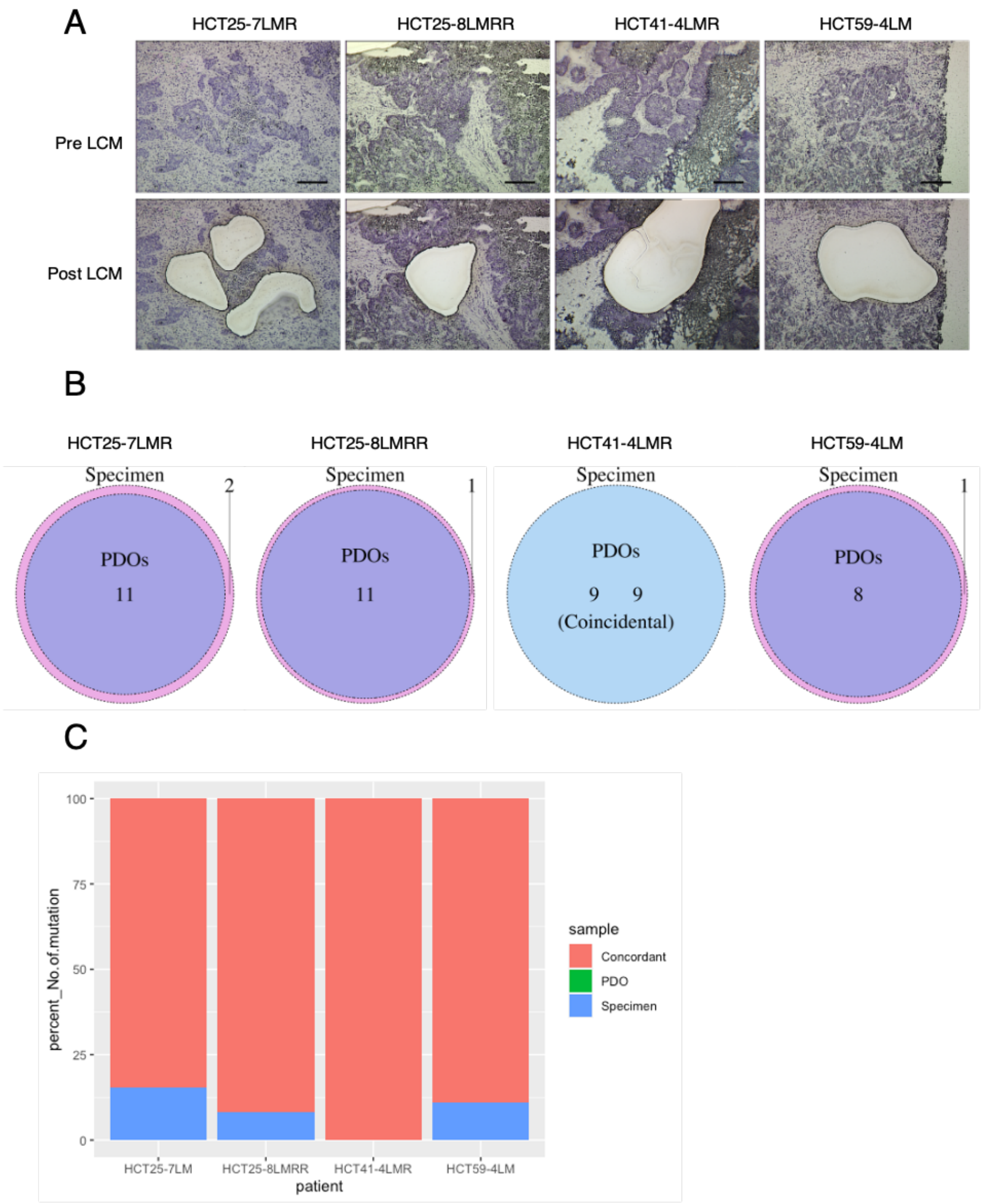

Supplementary Figure 2

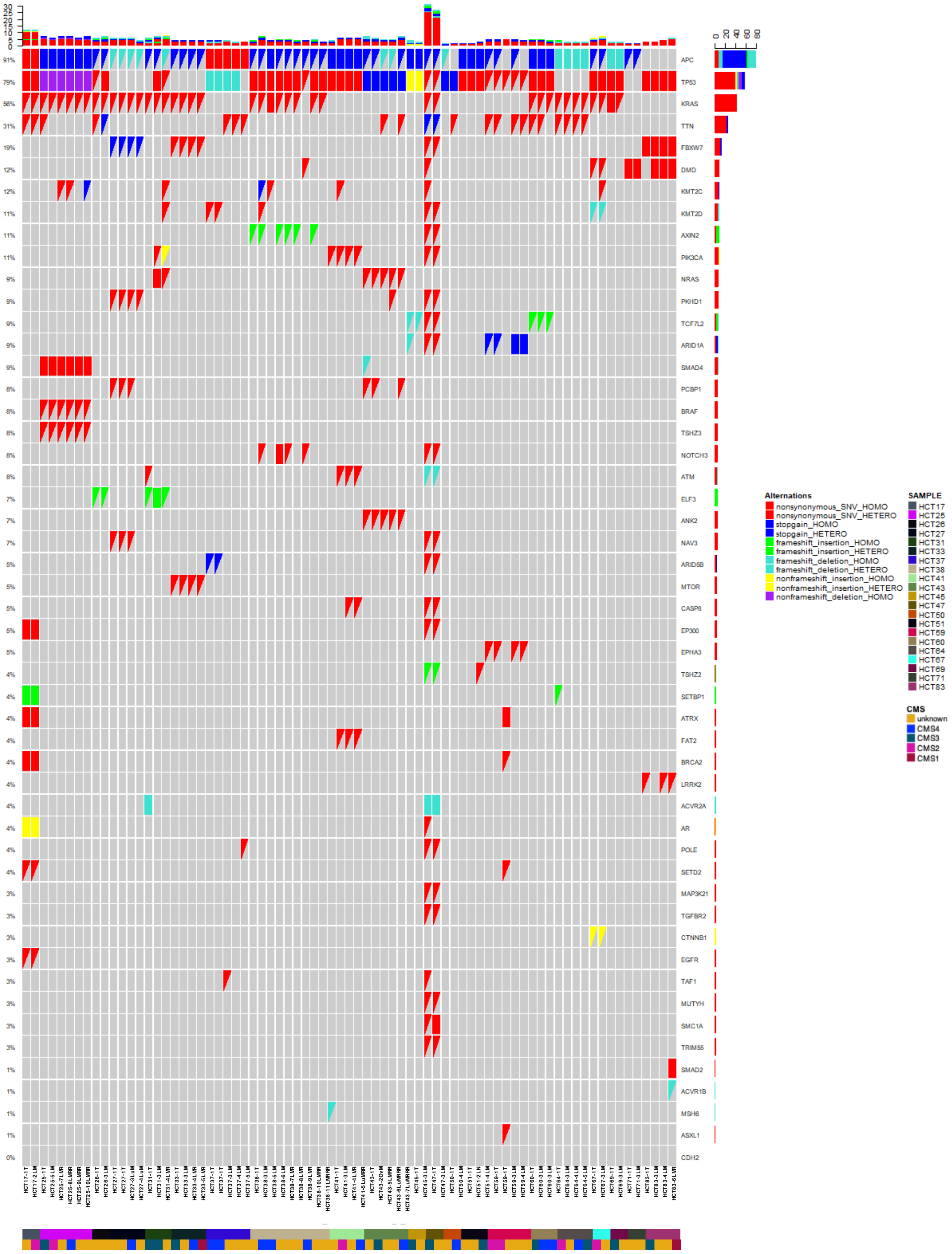

Supplementary Figure 3

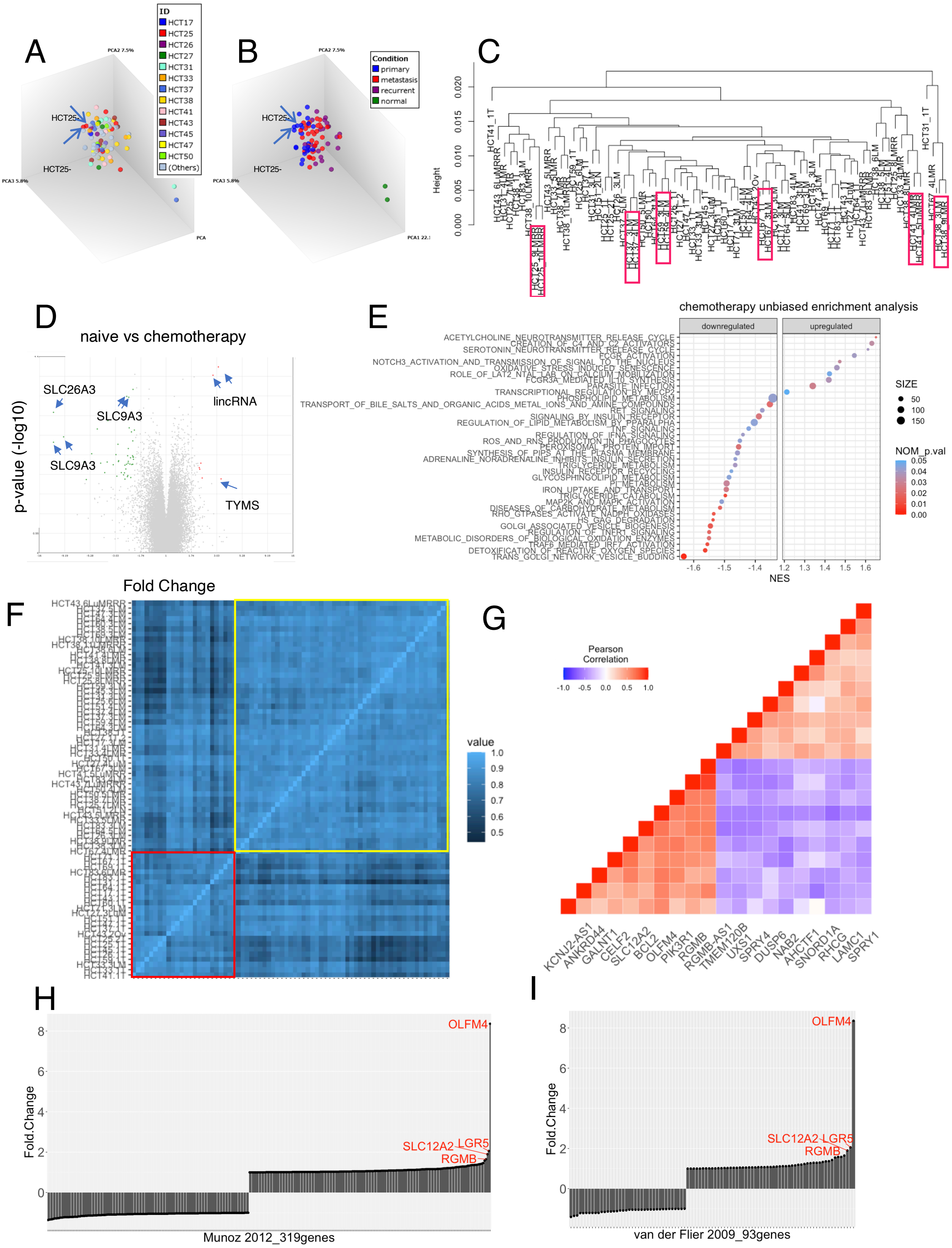

Supplementary Figure 4

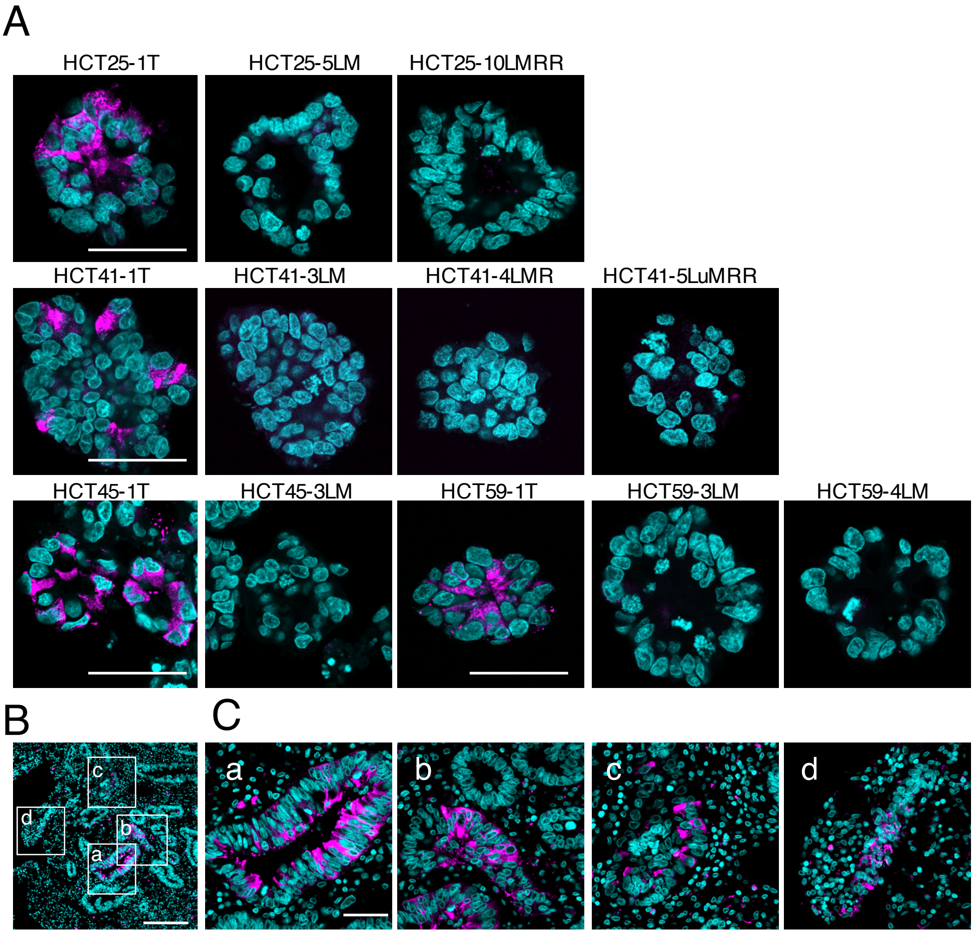

Supplementary Figure 5

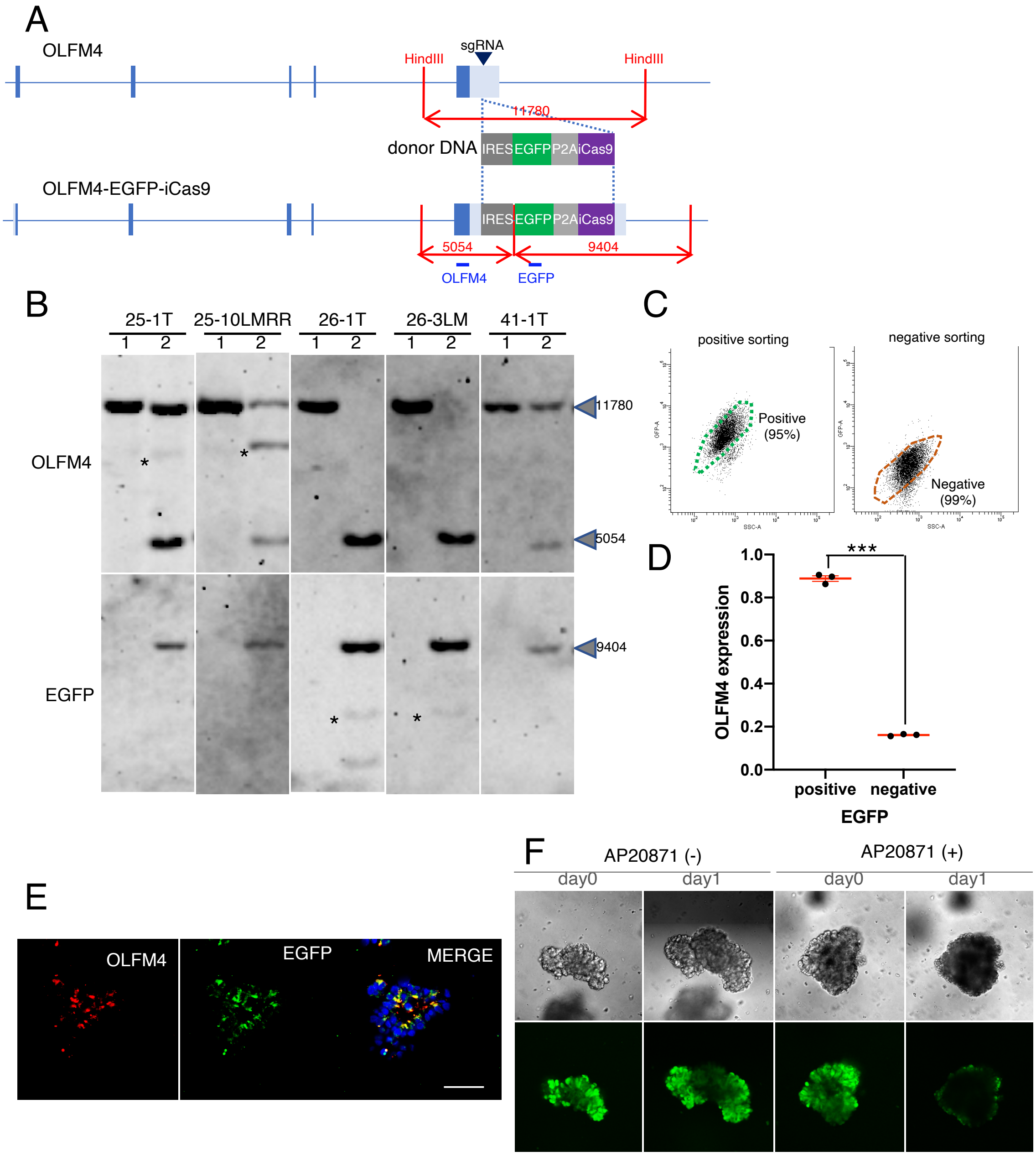

Supplementary Figure 6

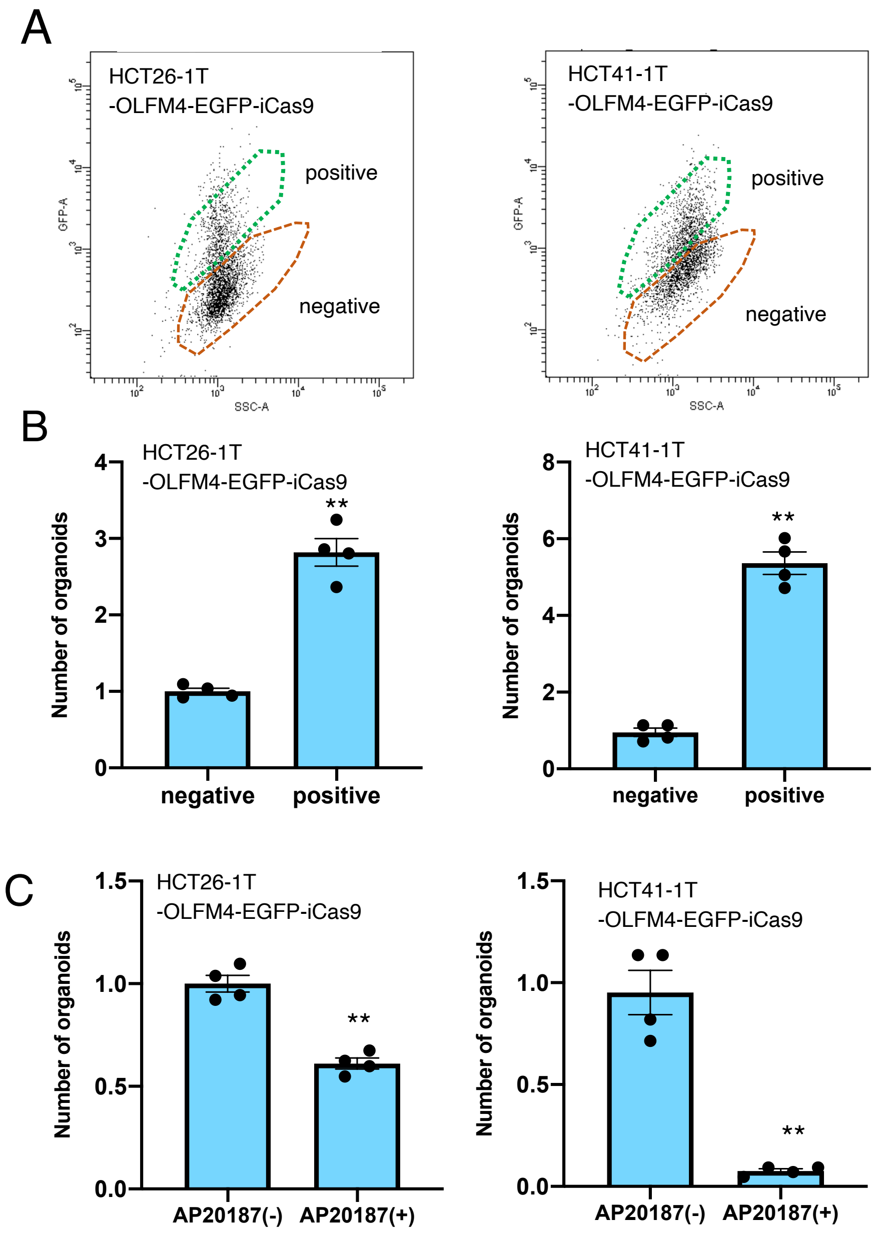

Supplementary Figure 7

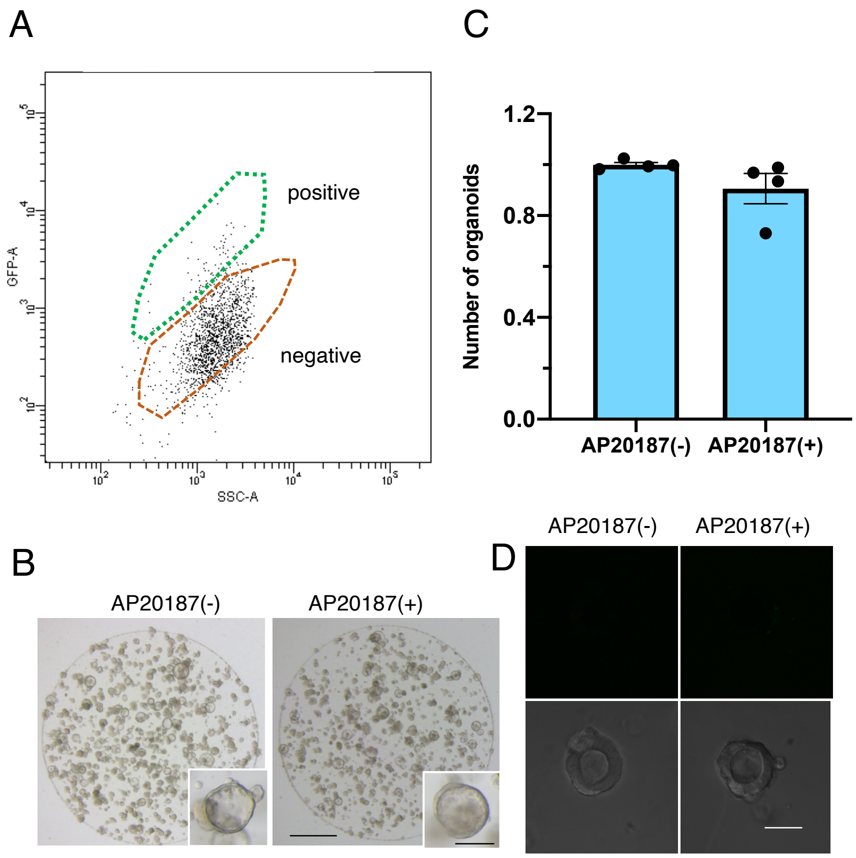

Supplementary Figure Legends

Supplementary Figure 1. Mutations in PDOs and corresponding surgical specimens. Related to Figure 1.

(A) Hematoxylin-stained frozen sections of surgical specimens. Images pre- and post-laser captured microdissection are shown. Bar=200µm

(B) Venn diagram showing the concordance of mutations shared between PDOs and the corresponding surgical specimen.

(C) Concordance of mutations detected in PDOs and corresponding surgical specimens. The bar graph represents the percentage of mutations that are detected only in PDOs (PDOs) and the corresponding surgical specimen (Specimen). Mutations detected in both samples are shown (Concordant).

Supplementary Figure 2. Genomic profiles of CRC PDOs. Related to Figure 1.

Overview of 69 mutations found in 71 PDOs. The nine most frequently mutated genes are listed, and the mutation frequencies are shown in the right row. The patients from which the PDOs were established and their CMS grouping are shown in the bottom column.

Supplementary Figure 3. Transcriptome analysis of PDOs. Related to Figure 1.

(A, B) Principal component analysis of PDOs. Colors represent the patients (A) and lesions (B) from which PDOs were established. Two PDOs established from normal mucosa are shown in green (B). Two primary PDOs derived from identical surgical specimens (HCT25-1T and -2T) are indicated by arrows. (C) Dendrograms (average distance method) based on Pearson correlation. The red boxes show PDO clusters derived from the same patients. (D) Volcano plot of comparative gene expression analysis between naïve and chemotherapy-treated PDOs. PDOs were cultured for five days, and microarray analysis was performed. Red dots represent genes exhibiting a significant difference (fold change > 2.0 and p-val<0.01). (E) Dot plot of GSEA results. The significantly activated and suppressed pathways derived from REACTOME are listed. The color of the dots represents the nominal P value, and the diameter represents the enriched gene count. (F) Correlation matrix of PDOs. The strength of the correlation is represented by color. Pearson's correlation analysis. The clusters mostly composed of

primary PDOs and metastatic/recurrent PDOs are shown by red and yellow frames, respectively. (G) Correlation matrix of gene expression. Ten genes most positively and negatively correlated with OLFM4 are shown in red and blue, respectively. Numbers represent correlation coefficients. (H, I) The expression of ISC signature genes reported by Munoz et al. (H) and van der Filer et al. (I) in PDOs is shown. The differential expression between primary and patient-matched PDOs is indicated by the fold change. The genes included in the metastasis signature are shown in red.

Supplementary Figure 4. Immunohistochemical analysis of OLFM4. Related to Figure 4.

(A) PDOs were fixed, and FFPE sections were stained with an anti-OLFM4 antibody (shown in magenta). Nuclei were visualized using DAPI (shown in cyan). Bar=50 μm. (B, C) Surgical specimens of HCT41-1T were fixed, and FFPE sections were stained with an anti-OLFM4 antibody (shown in magenta). Nuclei were visualized using DAPI (shown in cyan). Bar=200 μm. (C) High magnification of panel B. Bar=50 μm.

Supplementary Figure 5. Genome editing of PDOs. Related to Figures 5 to 7.

(A) An IRES-EGFP-P2A-iCas9 cassette was inserted into the 3' UTR of OLFM4. HindIII sites and the probes used for Southern blot analysis are shown in red and blue, respectively. The expected fragment sizes for each probe are shown in red. (B) Southern blot analysis of genome-edited PDOs. 1. Control PDOs; 2. Genome-edited PDOs. The probes and signal sizes are shown. Single allelic mutant organoids were obtained except in HCT26-1T and 3LM, which harbored biallelic insertions. (C) Fluorescence-activated cell sorting (FACS) chart of HCT25-1T and HCT25-1T cells harboring the IRES-EGFP-P2A-iCaspase9 cassette in the 3' UTR of the OLFM4 locus (HCT25-1T-OLFM4-EGFP-iCas9). The gates for EGFP-positive and EGFP-negative cells are shown in green and red, respectively. (D) RT-PCR analysis of OLFM4. EGFP-positive and EGFP-negative HCT25-1T-OLFM4-EGFP-P2A-iCaspase9 cells were isolated by FACS sorting, as shown in Figure S5C, and OLFM4 expression was analyzed by real-time RT-PCR. The data are shown as the mean and SD (shown in red). *** $P<0.001$ (unpaired t-test, N=3) (E) Immunofluorescence analysis using OLFM4 (shown in red) and EGFP (shown in green). Nuclei were visualized using DAPI. Bar=50 μm. (F) Ablation of $OLFM4^+$ cells. HCT25-1T/OLFM4-EGFP-iCas9 organoids were treated

with 2.5 nM AP20871 (AP20871(+)) or left untreated (AP20871(-)) for 1 day. Bar=50 μm.

Supplementary Figure 6. Organoid reconstruction efficiency from single cells. Related to Figure 5-6. (A) Fluorescence-activated cell sorting (FACS) chart of HCT26-1T and HCT41-1T harboring the IRES-EGFP-P2A-iCaspase9 cassette in the 3' UTR of the OLFM4 locus (HCT26-1T-OLFM4-EGFP-iCas9 and HCT41-1T-OLFM4-EGFP-iCas9, respectively). The gates for EGFP-positive and EGFP-negative cells are shown in green and red, respectively. (B) Organoid reconstruction efficiency from single cells. Flow-sorted single cells of HCT26-1T-OLFM4-iCas9 or HCT41-1T-OLFM4-iCas9 were cultured for 9 days, and the number of organoids was counted. The number of organoids relative to that of organoids generated from OLFM4$^-$ cells is shown. Data are shown as the mean and SD. **p<0.01(unpaired t-test, n=4 independent experiments). (C) AP20187 suppressed the growth of EGFP-negative cells. Flow-sorted EGFP-negative HCT26-1T-OLFM4_EGFP-iCas9 or HCT41-1T-OLFM4-iCas9 cells were cultured with or without AP20187 for 9 days. The number of organoids was counted and is shown as the relative number of organoids generated without AP20187. Data are shown as the mean and SD. **p<0.01(unpaired t-test, n=4 independent experiments).

Supplementary Figure 7. Generation of organoids from OLFM4$^-$ single cells in metastatic PDOs. Related to Figure 7. (A) FACS chart of HCT26-3LM-OLFM4-EGFP-iCas9. The gates for EGFP-positive and EGFP-negative cells are shown in green and red, respectively. (B) Reconstitution of organoids from EGFP-negative cells without (-) or with (+) AP20187. Bar=1000 μm, bar in inset=200 μm (C) The efficiency of organoid reconstitution. The number of organoids was counted using Fiji software, and the number of organoids when cultured with AP20187 relative to the number of organoids generated without AP20187 is shown. Data are shown as the mean and SD. **p<0.01(unpaired t-test, n=4 independent experiments). (D) Lack of EGFP expression in metastatic PDOs. Organoids were cultured for seven days and analyzed under a confocal microscope. Bar=50 μm.

Table S1. Clinical information of PDOs derived from stage IV CRC.

Table S2. Genes differentially expressed among distinct lesions.

Table S3. Expression of metastatic signature genes in PDOs derived from primary tumors and the patient matched metastatic lesions.

Table S4. Expression of ISC signatures (319 genes reported by Munoz et al. 2012) in PDOs derived from primary tumors and the patient matched metastatic lesions.

Table S5. Expression of ISC signatures (93 genes reported by van der Flier et al. 2009) in PDOs derived from primary tumors and the patient matched metastatic lesions.

Table S6. Top200 Differentially expressed genes in five clusters identified by scRNA-seq of CRC PDOs.

Table S7. Oligonucleotides used in this study.

Supplemental Experimental Procedures

Establishment of PDOs. Tumor samples were obtained from patients who provided informed consent, and all procedures were approved by the Research Ethics Board at the JFCR Cancer Institute (Tokyo, Japan). PDOs were established as previously described (Sakahara et al., 2019). Briefly, following surgical resection, tumors were enzymatically dissociated in digestion buffer (DMEM (Thermo Fisher Scientific) containing 0.0625% collagenase (Sigma-Aldrich), 0.125% dispase (Thermo Fisher Scientific) and 2.5% FBS and suspended in Matrigel (BD Bioscience), and 25 µl of this mixture was dispensed into each well of a culture plate. The organoids were cultured in ENR medium (basal medium supplemented with ENR (10 ng/ml EGF (Invitrogen), 10% Noggin conditioned medium and 1 µg/ml R-spondin-1 (R&D system)) at 37°C in 5% O_2.

Sequence analysis. Organoids were collected in Cell Recovery Solution (Corning). To analyze the sequence of surgical specimens, frozen tumor tissue sections were stained with hematoxylin, and tumor regions were excised by laser capture microdissection using a Leica LMD6000 microscope. Genomic DNA was extracted with a QIAamp DNA Mini Kit (Qiagen), and the DNA quality was analyzed using the Qubit system (Thermo Fisher Scientific). Two hundred nanograms of genomic DNA was used for library construction with a HaloPlex Kit (Agilent) according to the manufacturer's instructions. Target genes were selected as previously reported (Sakahara et al., 2019). Sequencing was performed using a MiSeq system (Illumina). Single-nucleotide variants (SNVs) and indels were called by the GATK (version 4.0.3) HaplotypeCaller (DePristo et al., 2011). The variants were filtered using GATK VariantFiltration with the following parameters: for SNV: --filter-expression "QD < 2.0 || FS > 60.0 || MQ < 40.0 || HaplotypeScore > 13.0 || MappingQualityRankSum < -12.5 || ReadPosRankSum < -8.0", for indel: --filter-expression "QD < 2.0 || FS > 200.0 || ReadPosRankSum < -20.0". The filtered variants were annotated with ANNOVER (Wang et al., 2010). After annotation, we extracted nonsynonymous SNVs and indels using our Perl script.
To extract somatic mutations, we excluded germline variants of NCBI dbSNP build 147 (Sherry et al., 2001) from the detected variants of tumor tissues and rescued known somatic mutations registered in the COSMIC database (version 70) (Forbes et al., 2017)

using our in-house Perl script. We also removed germline variants that were detected in normal tissue samples.

Microarray analysis. Organoids were collected in Matrigel Recovery Solution (Corning), and RNA was extracted using an RNeasy Micro Kit (Qiagen). RNA quality was validated using an Agilent Bioanalyzer. One hundred nanograms of RNA was used to prepare cRNA with a 3' IVT PLUS Reagent Kit (Affymetrix), and the cRNA was hybridized on the Human Transcriptome Array 2 (HTA2) (Affymetrix). Data were analyzed using Transcriptome Analysis Console (TAC) (Affymetrix). Analysis and visualization of the transcriptome data were performed in R (version 4.0) and RStudio (version 1.2). Clustering was carried out using the average method in the hclust function, and correlation analysis was performed with the Pearson method. The results were visualized using gglot2 (version 3.0.01).
Molecular subtyping was performed on the microarray transcriptome data in R (version 3.5.1) using the oligo package (version 1.46.0). After normalization using the RMA function, classification was performed using the CMScaller method with the default parameters to classify the CMS function (Eide et al., 2017).

Histochemical analysis. PDOs cultured in Matrigel for four to 6 days were extracted with Matrigel Recovery Solution, washed with PBS, fixed in 4% paraformaldehyde (PFA), held in 70% ethanol, and embedded in HitoGel (Thermo Fisher Scientific) before paraffin embedding. After dewaxing and rehydration via a standard procedure, antigen retrieval was performed by autoclaving in citrate buffer (pH 6.0) or by microwave treatment in TE buffer (pH 8.0). Surgical specimens were fixed in formalin and embedded in paraffin; the slides were incubated overnight at 4°C with primary anti-OLFM4 antibodies (D1E4M, Cell Signaling #14369) followed by Cy3-conjugated secondary antibodies (Merck Millipore, APC132).

scRNA-seq. PDOs were dissociated with Triple LE Express (Life Technology); undigested cell clusters were removed with a 40-μm cell strainer (Falcon). Single cells were washed twice with PBS. ScRNA-seq libraries were prepared using Chromium Single Cell 3' Solution (Reagent Kits version 1, 10x Genomics) according to the manufacturer's instructions. Briefly, using a Chromium Single Cell 3' Library, Gel

Bead & Multiplex Kit and Chip Kit (10x Genomics), Gel Bead-In-EMulsions (GEMs) constructed from isolated cells, Master Mix, Gel Beads and Partitioning Oil were created using the Chromium system (10x Genomics). After GEM generation, GEM-RT was performed, and the barcoded cDNA was cleaned and amplified. The amplified cDNAs were quantified using an Agilent 2100 Bioanalyzer High Sensitivity DNA Kit (Agilent). The cDNAs were sheared and size-selected for library construction; end repair, A-tailing, adaptor ligation and sample index PCR were also conducted. The constructed library was quantified using a Bioanalyzer.

The libraries were sequenced using a HiSeq2500 system (Illumina) according to the manufacturer's instructions. After sequencing analysis, fastq files were created using Cell Ranger (10x Genomics).

We filtered out cells that were 3x the mean absolute deviation (MAD) from the mean of the log coverage or 2x the MAD of the percentage of zeros. Genes without 1 count among at least 10 cells were filtered out, resulting in the removal of 4763 genes. Sample bias was corrected using the zero-inflated negative binomial model (ZINB-WaVE) (Risso et al., 2018).

Genome editing and in vitro analysis of PDOs. We used CRISPR/Cas9-mediated homology-independent targeted integration (HITI) to achieve efficient genome editing (Suzuki et al., 2016). sgRNA-expressing constructs were prepared by cloning a pair of annealed oligonucleotides into the BbsI site of pX330 (Addgene #422320). To construct a donor vector for OLFM4-EGFP-P2A-iCaspase9, the P2A-iCaspase9 fragment was amplified by PCR using pMSCV-F-del Casp9.IRES. GFP (Addgene #15567) was used as a template, and PvuI and PacI sites were created. The fragment was then cloned into the PacI site of pCMMP-MCS-IRES-eGFP (Addgene #36953), which was modified to disrupt the stop codon of the eGFP gene. The 20-bp sgRNA recognition sequence oligonucleotide was cloned into AgeI/NotI and PacI/NheI sites. The oligonucleotide sequences are listed in Table S7. The donor construct and sgRNA and Cas9 expression vector were electrophoresed using the piggyBac system as previously described (Fujii et al., 2015). The transfected organoids were selected with 2 μg/ml puromycin from 5 days after electroporation. Genomic DNA was prepared using lysis buffer (10 mM Tris-HCl, pH 8.0, 25 mM EDTA, 1% SDS, 150 mM NaCl, 100 μg/ml Proteinase K), followed by phenol-chloroform extraction and ethanol precipitation. Southern blot analysis was

performed using standard procedures and the DIG system (Roche). The primers used to prepare DIG probes are listed in Table S7.

For FACS analysis, PDOs were dissociated using Triple LE Express (Life Technology), and undigested cell clusters were removed with a 20-µm cell strainer (Falcon). The cells were washed twice with PBS supplemented with 0.2% BSA and 2 mM EDTA and stained with 7-aminoactinomycin D (BD Bioscience). Single cells were gated based on the SSC-H vs SSC-W profile. The cells were sorted using a 100-µm nozzle (Aria III, BD Bioscience), and $1x10^4$ cells were embedded in 25 µl of Matrigel and cultured in a 48-well plate. Images were analyzed using the cell counter plugin installed in Image J (version 2.0.0). Data are presented as the mean and SD (error bars) of four independent experiments. Expression of EGFP was analyzed via confocal scanning microscopy (Zeiss LSM880). To evaluate the reconstruction efficiency, pictures were taken using a stereomicroscope (Leica M165C), and the images were analyzed using the cell counter plugin installed in ImageJ (version 2.0.0). All data were derived from four independent experiments.

Supplemental References

DePristo, M.A., Banks, E., Poplin, R., Garimella, K.V., Maguire, J.R., Hartl, C., Philippakis, A.A., del Angel, G., Rivas, M.A., Hanna, M., *et al.* (2011). A framework for variation discovery and genotyping using next-generation DNA sequencing data. Nat Genet *43*, 491-498.

Eide, P.W., Bruun, J., Lothe, R.A., and Sveen, A. (2017). CMScaller: an R package for consensus molecular subtyping of colorectal cancer pre-clinical models. Scientific reports *7*, 16618.

Forbes, S.A., Beare, D., Boutselakis, H., Bamford, S., Bindal, N., Tate, J., Cole, C.G., Ward, S., Dawson, E., Ponting, L., *et al.* (2017). COSMIC: somatic cancer genetics at high-resolution. Nucleic Acids Res *45*, D777-D783.

Fujii, M., Matano, M., Nanki, K., and Sato, T. (2015). Efficient genetic engineering of human intestinal organoids using electroporation. Nat Protoc *10*, 1474-1485.

Risso, D., Perraudeau, F., Gribkova, S., Dudoit, S., and Vert, J.P. (2018). A general and flexible method for signal extraction from single-cell RNA-seq data. Nature communications *9*, 284.

Sakahara, M., Okamoto, T., Oyanagi, J., Takano, H., Natsume, Y., Yamanaka, H., Kusama, D., Fusejima, M., Tanaka, N., Mori, S.*, et al.* (2019). IFN/STAT signaling controls tumorigenesis and the drug response in colorectal cancer. Cancer Sci.

Sherry, S.T., Ward, M.H., Kholodov, M., Baker, J., Phan, L., Smigielski, E.M., and Sirotkin, K. (2001). dbSNP: the NCBI database of genetic variation. Nucleic Acids Res *29*, 308-311.

Suzuki, K., Tsunekawa, Y., Hernandez-Benitez, R., Wu, J., Zhu, J., Kim, E.J., Hatanaka, F., Yamamoto, M., Araoka, T., Li, Z.*, et al.* (2016). In vivo genome editing via CRISPR/Cas9 mediated homology-independent targeted integration. Nature *540*, 144-149.

Wang, K., Li, M., and Hakonarson, H. (2010). ANNOVAR: functional annotation of genetic variants from high-throughput sequencing data. Nucleic Acids Res *38*, e164.
